# Influence of individuals’ determinants including vaccine type on cellular and humoral responses to SARS-CoV-2 vaccination

**DOI:** 10.1038/s41541-024-00878-0

**Published:** 2024-05-22

**Authors:** Emma S. Chambers, Weigang Cai, Giulia Vivaldi, David A. Jolliffe, Natalia Perdek, Wenhao Li, Sian E. Faustini, Joseph M. Gibbons, Corinna Pade, Alex G. Richter, Anna K. Coussens, Adrian R. Martineau

**Affiliations:** 1 Centre for Immunobiology, Blizard Institute, Barts and the London School of Medicine and Dentistry, Queen Mary University of London, London, E1 2AT UK; 2 Institute of Immunology and Immunotherapy, College of Medical and Dental Sciences, University of Birmingham, Birmingham, B15 2TT UK; 3 Centre for Genomics and Child Health, Blizard Institute, Barts and the London School of Medicine and Dentristry, Queen Mary University of London, London, E1 2AT UK; 4 Infectious Diseases and Immune Defence Division, Walter and Eliza Hall Institute of Medical Research, Parkville, VIC 3052 Australia; 5 Centre for Infectious Diseases Research in Africa, Institute of Infectious Disease and Molecular Medicine, University of Cape Town, Cape Town, 7925 South Africa

**Keywords:** RNA vaccines, Risk factors

## Abstract

Vaccine development targeting SARS-CoV-2 in 2020 was of critical importance in reducing COVID-19 severity and mortality. In the U.K. during the initial roll-out most individuals either received two doses of Pfizer COVID-19 vaccine (BNT162b2) or the adenovirus-based vaccine from Oxford/AstraZeneca (ChAdOx1-nCoV-19). There are conflicting data as to the impact of age, sex and body habitus on cellular and humoral responses to vaccination, and most studies in this area have focused on determinants of mRNA vaccine immunogenicity. Here, we studied a cohort of participants in a population-based longitudinal study (COVIDENCE UK) to determine the influence of age, sex, body mass index (BMI) and pre-vaccination anti-Spike (anti-S) antibody status on vaccine-induced humoral and cellular immune responses to two doses of BNT162b2 or ChAdOx-n-CoV-19 vaccination. Younger age and pre-vaccination anti-S seropositivity were both associated with stronger antibody responses to vaccination. BNT162b2 generated higher neutralising and anti-S antibody titres to vaccination than ChAdOx1-nCoV-19, but cellular responses to the two vaccines were no different. Irrespective of vaccine type, increasing age was also associated with decreased frequency of cytokine double-positive CD4+T cells. Increasing BMI was associated with reduced frequency of SARS-CoV-2-specific TNF+CD8% T cells for both vaccines. Together, our findings demonstrate that increasing age and BMI are associated with attenuated cellular and humoral responses to SARS-CoV-2 vaccination. Whilst both vaccines induced T cell responses, BNT162b2 induced significantly elevated humoral immune response as compared to ChAdOx-n-CoV-19.

## Introduction

SARS-CoV-2 is the causative agent of coronavirus disease 2019 (COVID-19). As of February 2024 SARS-CoV-2 has infected over 774 million people and is responsible for over 6.9 million deaths worldwide^1^, however this is likely to be an under-estimation as excess mortality due to COVID-19 in April 2022 was estimated to be 18 million people^2^. SARS-CoV-2 also causes the debilitating illness, long-COVID, which affects around 10% of those infected^3^. Due to the profound morbidity and mortality caused by SARS-CoV-2, the development of vaccines in 2020 was of critical importance. In the U.K. three vaccines were licenced for use by the start of 2021. These include the mRNA vaccines from Pfizer (BNT162b2) and Moderna (mRNA-1273) and the adenovirus-based vaccine from AstraZeneca/Oxford (ChAdOx1-nCoV-19)^4^. In 2020-2021 during the initial roll-out of COVID-19 vaccination in the U.K., most individuals received a primary course comprising two doses of BNT162b2 or ChAdOx1-nCoV-19^5,6^.

Individuals who are male, older in age, or with high Body Mass Index (BMI) are at increased risk of morbidity and mortality from COVID-19^7,8^—therefore, studies have focussed on vaccine immunogenicity in these at risk populations. Increasing age has been associated with reduced IgG and neutralising antibody titre after two BNT162b2 vaccine doses^9,10^. In contrast, a study assessing both BNT162b2 or ChAdOx1 immunogenicity showed no significant difference in humoral or cellular immune response after vaccination in those ≥80 years as compared to those <80 years^11^. The published BMI studies show conflicting results, with either no effect^10,12^, decreased^13,14^ or increased^15^ humoral BNT162b2 vaccine immunogenicity observed with increased BMI. Few studies have shown any influence of sex on vaccine efficacy—of those mentioned above, only one identified that females have a higher anti-Spike IgG antibody titre post-BNT162b2 vaccination^10^.

There are relatively few studies investigating the cellular and humoral immune response to both BNT162b2 and ChAdOx1-nCoV-19 in those individuals at risk, with most studies focussing on the impact of individual demographics on mRNA vaccine efficacy. There is also a lack of studies assessing individuals’ determinants on cellular versus humoral immunity. Here, we assessed a population-based cohort from the COVIDENCE study^16^, who had either two doses of BNT162b2 or ChAdOx-n-CoV-19 to determine the influence of individual demographic variables such as age, sex, BMI, and pre-vaccination IgG/A/M anti-Spike seropositivity on SARS-CoV-2 antibody and antigen-specific CD4+ and CD8+ T cell response, bulk T cell functional phenotypes and antigen-induced whole blood cytokine secretion following to the first two doses of the vaccines BNT162b2 and ChAdOx-n-CoV-19.

## Results

### Identification of individual correlates of immunogenicity

Characteristics of the 115 participants with humoral and cellular data included in the analyses are presented in Table 1. The median age was 66.4 years (IQR 61.0–68.9), 47 (41%) males and 68 (59%) females, 57 (49.6%) had a BMI less than 25, 45 (39.1%) had a BMI of 25–30 and a further 13 (11.3%) had a BMI of greater than 30. Of the participants 77 (66.9%) received ChAdOx1 and 38 (33.0%) received BNT162b2. When individuals were separated according to vaccine type received, the individual characteristics were similar (Supplementary Table 1). To identify demographic and technical factors associated with humoral and cellular responses to COVID-19 vaccination we first performed univariate analysis of ten factors: age, sex, ethnicity, general health category, vaccine type, number of days between 1st and 2nd vaccination [inter-vaccine days], days post second vaccine, pre-vaccine SARS-CoV-2 serostatus, BMI value, and BMI category. This analysis identified seven factors, which had a significant association with cellular and/or humoral immune responses: age, sex, pre-vaccine SARS-CoV-2 serostatus, BMI category, vaccine type, inter-vaccine days, and days post second vaccine (Supplementary Tables 2a–i). These seven factors were adjusted for in subsequent analyses to identify independent associations.

**Table 1:** Participant characteristics (*n* = 115)

Characteristic		
Age	Median age, years (IQR)	66.4 (61.0–68.9)
	Age range, years	23.5–78.6
Sex, *N* (%)	Male	47 (40.9%)
	Female	68 (59.1%)
Ethnicity, *N* (%)	White	107 (93.0%)
	South Asian	1 (0.9%)
	Black/African/Caribbean/Black British	0
	Mixed/Multiple/Other	7 (6.1%)
Body mass index, kg/m^2^, *N* (%)	<25	57 (49.6%)
	25–30	45 (39.1%)
	>30	13 (11.3%)
Highest educational level attained, *N* (%)^3^	Primary/Secondary	9 (7.8%)
	Higher/Further (A levels)	9 (7.8%)
	College	55 (47.8%)
	Post–graduate	42 (36.5%)
Quantiles of index of multiple deprivation, *N* (%)	Q1 (most deprived)	19 (16.5%)
	Q2	21 (18.3%)
	Q3	38 (33.0%)
	Q4 (least deprived)	37 (32.2%)
Tobacco smoking, *N* (%)	Non–current/never smoker	111 (96.5%)
	Current smoker	4 (3.5%)
Alcohol consumption/week, units, *N* (%)	None	21 (18.3%)
	1–7	43 (37.4%)
	8–14	26 (22.6%)
	15–21	16 (13.9%)
	22–28	8 (7.0%)
	>28	1 (0.9%)
Self-assessed general health, *N* (%)	Excellent	29 (25.4%)
	Very good	43 (37.7%)
	Good	31 (27.2%)
	Fair	11 (9.6%)
	Poor	0
Pre-vaccination anti-S IgG/A/M serostatus, *N* (%)	Negative	100 (81.7%)
	Positive	15 (12.2%)
Type of vaccine received for primary course, *N* (%)	2 x ChAdOx1	77 (66.9%)
	2 x BNT162b2	38 (33.0%)
Median inter-dose interval, weeks (IQR)		11.0 (10.0–11.3)
Median time from date of second vaccine dose to date of sampling, weeks (IQR)		11.9 (10.0–14.6)

*IQR* inter-quartile range, *s.d.* standard deviation, *Ig* Immunoglobulin. Self-assessed general health is in 115 participants, and median time from date of second vaccine was measured in 112 participants.

### Immune correlates with anti-Spike and SARS-CoV-2 neutralising antibody titres after SARS-CoV-2 vaccination

**Fig. 1:**
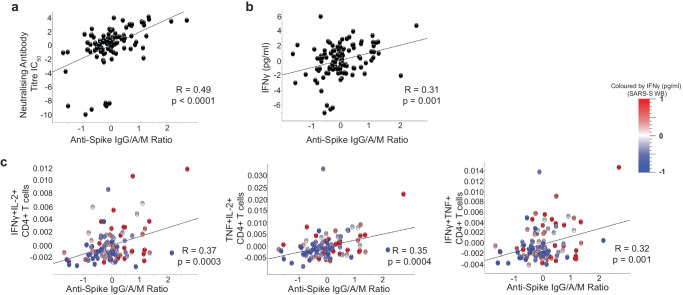
Humoral and cellular correlates of anti-S IgG/A/M antibody ratio after COVID-19 vaccination. Correlation between post-COVID-19 vaccine anti-S IgG/A/M antibody ratio and **a**, neutralising antibody titre IC_50_, **b**, whole blood IFNγ production after S peptide stimulation and **c**, percent of cytokine positive CD4+ T after PBMC stimulation with S peptide as determined by intracellular cytokine staining. Coloured according to IFNγ production from S peptide-stimulated whole blood (SARS-S WB). Data presented on x and y axes are normalised including log_2_ transformation and adjusted for the baseline and post-vaccination covariates (age, sex, BMI category, pre-vaccine SARS-CoV-2 serostatus, vaccine type, vitamin D randomisation, inter-vaccine days, and days post second vaccine), p values derived using the quadratic regression for general linear models with adjustments for covariates, all *q* < 0.01. Trend line indicates Pearson correlation (R-statistic).

First, we investigated whether there was any relationship between humoral and cellular immune responses we measured and post-vaccine anti-Spike and neutralising titres, adjusting for the seven baseline and post-vaccination covariates identified in univariate analyses described above. Post-vaccination titres of anti-Spike combined IgG/A/M antibody ratio responses were identified to be associated with neutralising antibody concentrations, three spike-specific CD4+T cell phenotypes and spike-stimulation induced IFNγ secretion (Fig. 1, Supplementary Table 3; quadratic regression for general linear models with adjustment for covariates). In addition, neutralising antibody concentrations significantly correlated with SARS-CoV-2-specific TNF+CD8+ T cell frequency (*p* = 0.0003, Supplementary Table 4; quadratic regression for general linear models with adjustment for covariates). As expected, post-vaccination titres of neutralising and anti-Spike IgG/A/M antibody ratio correlated positively with each other (*R* = 0.47, *p* < 0.0001, Fig. 1a; Pearson correlation and quadratic regression for general linear models with adjustment for covariates respectively). In addition, anti-S IgG/A/M antibody ratio correlated positively with IFNy secretion from SARS-CoV-2 peptide-stimulated whole blood (*R* = 0.31, *p* = 0.001; Fig. 1b; Pearson correlation and quadratic regression for general linear models with adjustment for covariates respectively). The three spike-specific CD4+T cell populations from PBMC SARS-CoV-2 peptide-stimulated cultures that positively correlated with anti-Spike IgG/A/M antibody ratio post-vaccination were all double-positive cytokine producers: IFN-γ+IL2+ (*R* = 0.37, *p* = 0.0003; Pearson correlation and quadratic regression for general linear models with adjustment for covariates respectively); TNF+IL-2+ (*R* = 0.35, *p* = 0.0004; Pearson correlation and quadratic regression for general linear models with adjustment for covariates respectively); IFN-γ+TNF+ (*R* = 0.32, *p* = 0.001; Pearson correlation and quadratic regression for general linear models with adjustment for covariates respectively) (Fig. 1c). There was also a trend (*q* = 0.1) for a positive correlation between anti-Spike IgG/A/M and SARS-CoV-2 peptide-specific CD4+IL-2 single positive T cells (Supplementary Table 3; quadratic regression for general linear models with adjustment for covariates). Significant correlations were maintained between anti-Spike combined IgG/A/M antibody and neutralising antibody concentrations and spike-stimulation induced IFNγ secretion when not including vaccine type as a covariate adjustment (*R* = 0.56; Pearson correlation and Supplementary Fig. 4).

### Influence of pre-vaccination anti-S IgG/A/M antibody ratio on post-vaccine humoral and cellular responses

**Fig. 2:**
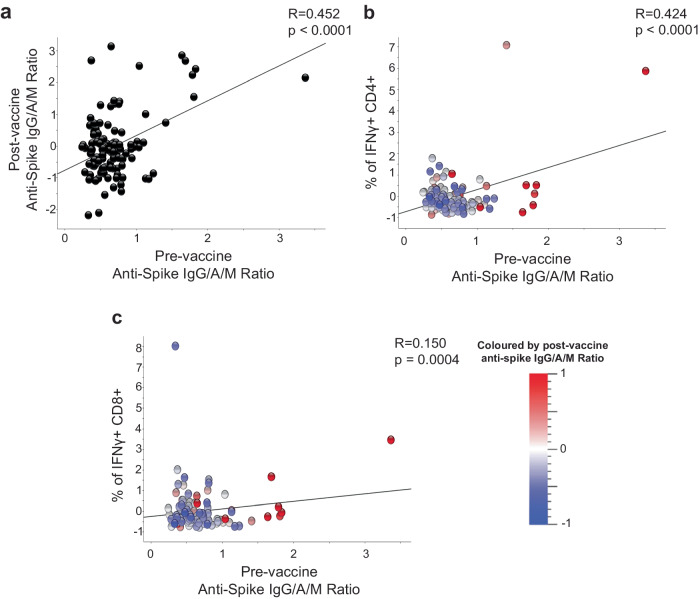
Humoral and cellular responses post-vaccination associated with pre-vaccination and anti-S IgG/A/M antibody ratio. Correlation between pre-COVID-19 vaccine anti-S IgG/A/M antibody ratio and post-COVID-19 vaccine. **a** anti-S IgG/A/M antibody ratio, and frequency of IFNy+ **b**, CD4+ and **c**, CD8+ T cells after S peptide stimulation, **b**, **c** coloured according to anti-S IgG/A/M antibody ratio normalised to mean 0 and variance ±1. Data presented on x and y axes are normalised including log2 transformation and adjusted for the baseline and post-vaccination covariates (age, sex, BMI category, vaccine type, vitamin D randomisation, inter-vaccine days, and days post second vaccine), *p* value derived using thequadratic regression for general linear models with adjustments for covariates, all *q* < 0.01. Trend line indicates Pearson correlation (R-statistic).

We next determined whether there was a relationship between pre-vaccine anti-Spike IgG/A/M antibody ratio on post-vaccine humoral and cellular responses (Supplementary Table 5), including adjustment for baseline and post-vaccination covariates (age, sex, BMI category, vitamin D randomisation, vaccine type, inter-vaccine days, and days post second vaccine). Pre-vaccine anti-Spike IgG/A/M antibody ratios were significantly correlated with post-vaccine anti-Spike IgG/A/M antibody ratios (*p* < 0.001; Pearson correlation and quadratic regression for general linear models with adjustment for covariates respectively) (Fig. 2a), frequency of IFNγ+CD4+ and IFNγ+CD8 + SARS-CoV-2-specific T cells (*p* < 0.001; quadratic regression for general linear models with adjustment for covariates) (Fig. 2b, c) and frequency of effector memory CD4+T cells in unstimulated PBMC (*p* = 0.006; Supplementary Table 5; quadratic regression for general linear models with adjustment for covariates). Comparing those who were considered seropositive (anti-Spike IgG/A/M antibody ratio ≥1) vs seronegative pre-vaccination, post-vaccine anti-Spike IgG/A/M antibody ratios and frequency of IFNγ+CD4+SARS-CoV-2-specific T cells remained significantly different (*p* < 0.001) (Supplementary Table 6; t-test for general linear models with adjustment for covariates). Together, these data demonstrate that individuals with baseline seropositivity due to prior SARS-CoV-2 infection had a stronger absolute cellular immune response following SARS-CoV-2 vaccination than those who were anti-S seronegative. These responses will be a combination of the original infection induced T cell memory and that expanded by the vaccination, as baseline PBMC were not taken, T cell expansion could not be assessed.

### Influence of vaccine type, inter-dose interval and time from vaccination to blood sampling on post-vaccine immune responses

**Fig. 3:**
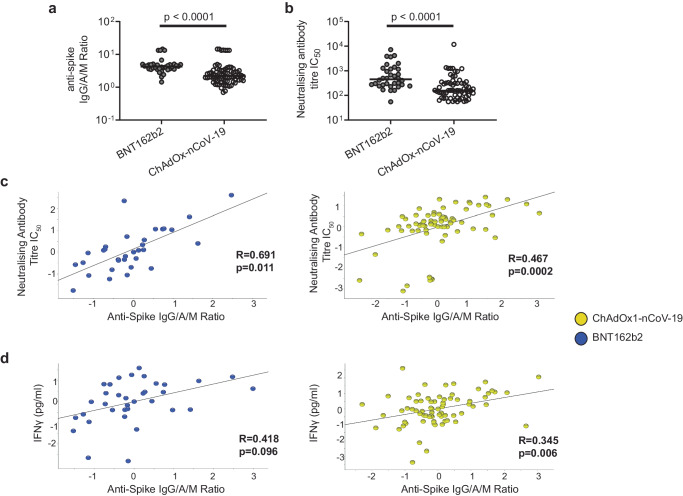
Post-vaccination antibody titres and post-vaccination cellular and humoral correlations by vaccine type. Cumulative data showing **a** anti-Spike IgG/A/M antibody ratio and **b** neutralising antibody titre IC_50_ in participants post-vaccination with either BNT162b2 or ChAdOx1-nCoV-19. Line indicated Median. Data points are plotted without covariate adjustment. Correlations plotted separately for participants who received either BNT162b2 (blue) or ChAdOx1-nCoV-19 (yellow) comparing post-vaccination anti-S IgG/A/M antibody ratio, and post-vaccination. **c** neutralising antibody (NAB) titre IC_50_. **d** whole blood IFNγ production after S peptide stimulation. Data presented on x and y axes are normalised including log_2_ transformation and adjusted for the baseline and post-vaccination covariates (age, sex, BMI category, pre-vaccine SARS-CoV-2 serostatus, vitamin D randomisation, inter-vaccine days, and days post second vaccine), *p* value derived using t-test (**a** and **b**) or quadratic regression (**c** and **d**) for general linear models with adjustment for covariates. Trend line indicates Pearson correlation (R-statistic).

Having identified cellular and humoral correlates of post-vaccination anti-S and neutralising antibody titre, irrespective of vaccine type, we next investigated whether there were any differences in cellular and humoral correlates between those who received BNT162b2 and ChAdOx1-nCoV-19. Adjusting for the other baseline and post-vaccination covariates, as above, we found BNT162b2 induced significantly higher anti-S IgG/A/M antibody ratio and neutralising antibody titres compared to ChAdOx1-nCoV-19 (Fig. 3a, b, Supplementary Table 7; t-test for general linear models with adjustment for covariates). There were, however, no significant differences in unstimulated or antigen-stimulated cellular responses that we measured in PBMC or whole blood between those who received BNT162b2 compared with those who received ChAdOx1-nCoV-19 (Supplementary Table 7). Separately analysing post-vaccination anti-S IgG/A/M antibody ratio correlations for each vaccine type, BNT162b2 had a stronger correlation compared to ChAdOx1-nCoV-19 with neutralising antibody titres (*R* = 0.691 vs *R* = 0.467, respectively; Pearson correlation) (Fig. 3c) and spike-stimulation induced IFNγ secretion (*R* = 0.418 vs *R* = 0.345, respectively) (Fig. 3d), although BNT162b2 Pearsons correlations were less significant due to smaller sample size compared to ChAdOx1-nCoV-19. Post-vaccination anti-S IgG/A/M antibody ratio correlations with the three polyfunctional spike-specific CD4+T cell populations previously identified, irrespective of vaccine type (Fig. 1c), showed stronger correlation for BNT162b2 with TNF+IL-2+CD4+T cells (*R* = 0.51, vs *R* = 0.31 ChAdOx1-nCoV-19; Pearson correlation) and IFN-γ+IL2 + CD4+T cells (*R* = 0.39, vs *R* = 0.35 ChAdOx1-nCoV-19; Pearson correlation); whereas ChAdOx1-nCoV-19 had stronger for IFN-γ+TNF+CD4+T cells (*R* = 0.33, vs *R* = 0.14 BNT162b2; Pearson correlation) (Supplementary Fig. 5).

When analysing whether there was an impact of the number of days between vaccinations and measured immune responses, we only identified a trend for the frequency of SARS-CoV-2 peptide-specific TNF+IFNγ+CD4+T cells and TNF+CD8+ T cells (*p* ≤ 0.007; *q* = 0.16) when adjusting for the other baseline and post-vaccination covariates (Supplementary Table 8; quadratic regression for general linear models with adjustment for covariates). However, we did find that the delay from the date of the second vaccine dose to the date of blood draw was positively correlated with the level of SARS-CoV-2 peptide-induced whole blood secretion of IL-6, IL-8 and TNF (*p* ≤ 0.0004), and a trend for negative correlation with Neutralising antibody titres (*p* = 0.01; *q* = 0.14) (Supplementary Table 9; linear regression for general linear models with adjustment for covariates).

### Increasing age significantly altered the immune response to vaccination

**Fig. 4:**
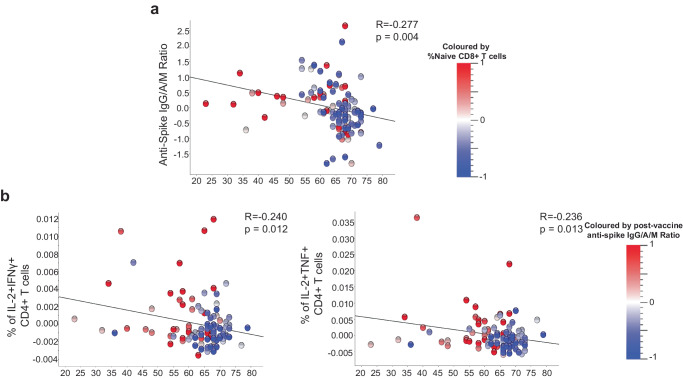
Relationship between post-vaccination anti-S IgG/A/M antibody ratio, percent naive CD8+ T cells, and age. **a** Correlation between age and post-vaccine anti-Spike IgG/A/M antibody ratio, coloured according to frequency of naive (CD45RA+CD27+) CD8+ T cells present in the peripheral blood of the individuals. **b** percent of cytokine positive CD4+T after PBMC stimulation with S peptide as determined by intracellular cytokine staining, coloured according to anti-S IgG/A/M antibody ratio normalised to mean 0 and variance ±1. Data presented on y axis is normalised including log_2_ transformation and adjustment for baseline and post-vaccination covariates (sex, BMI category, pre-vaccine SARS-CoV-2 serostatus, vaccine type, vitamin D randomisation, inter-vaccine days, and days post second vaccine), *p* values derived using linear regression for general linear models with adjustments for the same covariates, all *q* < 0.1. Trend line indicates Pearson correlation (R-statistic).

Having determined the vaccine type and timing variables independently associated with vaccine-induced humoral and cellular immune responses, we next analyzed demographic correlates adjusting for all other baseline and post-vaccination covariates as previously. We found that increasing age was independently associated with lower anti-S antibody titres post-vaccination (*R* = −0.277, all participants adjusting for vaccine type; Pearson correlation) (Fig. 4a). There was no difference when analysing each vaccine type independently (*R* = −0.26, BNT162b2; *R* = −0.27, ChAdOx1-nCoV-19, Supplementary Fig. 6A; Pearson correlation). However, due to lower number of individuals who received BNT162b2, this did not reach statistical significance. For all participants, the frequency of polyfunctional spike-specific CD4+T cells from PBMC SARS-CoV-2 peptide-stimulated cultures post-vaccination also negatively correlated with increasing age for IFN-γ+IL-2+ (*R* = −0.24, *p* = 0.012; Pearson correlation and linear regression for general linear models with adjustment for covariates respectively) and TNF+IL-2+CD4+T cells (*R* = −0.24, *p* = 0.013; Pearson correlationand linear regression for general linear models with adjustment for covariates respectively) (Supplementary Table 10; and Fig. 4b). Supplementary Fig. 6B, C shows the same analysis separating by vaccine type, ChAdOx1-nCoV-19 had a stronger negative correlation for both CD4+T cell populations with age, compared to BNT162b2 (IFN-γ+IL-2+, *R* = −0.34 vs *R* = −0.15 and TNF+IL-2+, *R* = −0.32 vs −0.14; Pearson correlation), although the larger and younger age range of those who received ChAdOx1-nCoV-19 may have improved the strength of correlation for ChAdOx1-nCoV-19. As would be expected, we also found increasing age was independently associated with lower frequency of naive CD8+ T cells (*p* < 0.0001) and higher frequency of CD8 + EM (*p* = 0.004) and EMRA T cells (*p* = 0.004). These data collectively show that increasing age was associated with reduced humoral immunity associated with reduced double-positive cytokine-producing spike-specific CD4+T cells, and vaccine type having no or only minor effect on these age-related differences.

### Influence of BMI and gender on post-vaccine immune responses

Finally, we tested for associations between sex and BMI with SARS-CoV-2 cellular and humoral immunity, with analyses adjusted for baseline and post-vaccination co-variates as previously. We found a highly significant lower frequency of SARS-CoV-2-specific TNF+CD4+ and CD8+ T cells (*p* < 0.0001) correlated with increasing BMI category (Supplementary Table 11; ANOVA for general linear models with adjustment for covariates), as well as trends of lower SARS-CoV-2-specific IL-2+CD4+T cells and higher CRP (*p* ≤ 0.014, *q* < 0.156). There was no significant association between higher BMI category and anti-Spike or neutralising antibodies. (Supplementary Table 11; ANOVA for general linear models with adjustment for covariates), despite our finding that the level of neutralising antibodies significantly correlated with SARS-CoV-2-specific TNF+CD8+ T cells (Supplementary Table 5), which were decreased with increasing BMI. This remained true if we analysed each vaccine type separately.

When analysing associations between sex immune correlates, the most significant difference was in our control assay with higher LPS induced IL-6 secretion in whole blood stimulated plasma (*p* < 0.0001). Males also had a higher frequency of CD8+ (*p* = 0.0001) and CD4+ (*p* = 0.011) EM T cells, whilst females had a higher frequency of naive CD4+ (*p* = 0.012) and CD8+ (*p* = 0.002) T cells (Supplementary Table 12; *t* test for general linear models with adjustment for covariates). However, these differences did not impact upon SARS-CoV-2-specific cellular or humoral responses post-vaccination between males and females with no significant differences observed (Supplementary Table 11). Collectively these data show sex had no impact on SARS-CoV-2 vaccine immunogenicity, whilst BMI had significant effects on single cytokine producing SARS-COV-2-specific T cell functions we independently identified to be associated with SARS-CoV-2 neutralising antibody concentrations post-vaccination.

## Discussion

In this study, we observed that there was a significant positive correlation between humoral and cellular SARS-CoV-2-specific immunity post vaccination which were modified by increasing age and BMI but not sex of individuals. The levels of anti-Spike IgG/M/A and SARS-CoV-2 neutralising antibodies were significantly correlated with SARS-CoV-2 induced cytokine secreting double-positive CD4+T cells or TNF+CD8+ T cells, respectively. As expected, those individuals who were seropositive prior to vaccination had the largest humoral and cellular immune response following SARS-CoV-2 vaccination, irrespective of vaccine type. We observed that there were significantly higher antibody titres and a stronger correlation between anti-Spike and neutralising antibody titres in participants that received BNT162b2 as compared to those that received ChAdOx1-nCoV-19. However, no unique antigen-specific cellular immunity differences were observed post-SARS-CoV-2 vaccination that explained these vaccine immunogenicity differences although there were differences in the strength of correlation with the frequency of distinct spike-specific CD4+T cell populations. By contrast, no impact of biological sex was observed for SARS-CoV-2 vaccine cellular and humoral immunity, despite cellular functional phenotype differences identified.

Previous studies have observed that BNT162b2 was more efficacious as a vaccine as compared to ChAdOx1-nCoV-19 at reducing infections and hospital admissions^17–19^. We observed that there were increased anti-S IgG/A/M antibody ratio and neutralising antibody titres with BNT162b2 as compared to ChAdOx1-nCoV-19, in line with previous observations^11,15^. Interestingly, whilst we did not observe any unique SARS-CoV-2 antigen-specific T cell populations induced by the different vaccines, BNT162b2 induced anti-Spike IgG/M/A titres had the strongest correlation with Spike-specific TNF+IL-2+CD4+T cells, whereas the strongest correlation for ChAdOx1-nCoV-19 was for TNF+IFN-γ+CD4+T cells. It is important to note that both vaccines still resulted in a good humoral and cellular immune response in the participants in this study. In line with the current literature^15,20^, we observed that there was increased COVID-19 vaccine immunogenicity in individuals that have had a prior infection with SARS-CoV-2. This boost in immunogenicity is believed to be due to the phenomenon of hybrid vigour immunity^21^.

It has been shown that older adults have reduced vaccine efficacy to influenza and shingles vaccine^22,23^, which is believed to be in part to immunosenescence and inflammageing^24,25^. This was particularly worrying at the start of the pandemic as older adults had increased morbidity and mortality from COVID-19^8^. Therefore, when the vaccines were developed, studies were performed to assess COVID-19 vaccine efficacy in older adults—the resulting studies were conflicting with results demonstrating similar vaccine efficacy (as determine by infection events and hospitalisation) by age or reduced vaccine efficacy. In this study we observed that increasing age was associated with reduced post-vaccine anti-S antibody titre, in line with other studies which have shown reduced responses post-SARS-CoV-2 vaccination^10,26^, although older age has been associated with lower risk of breakthrough infection^19^. In our study, our older adults were not as old as (oldest individual 78 years old) previous studies into ageing where the age range was 80-86 years old^11^, suggesting that age-associated decline in immunogenicity is linear and may start declining in middle-age. This reduced antibody response with increasing age has been shown to be alleviated after SARS-CoV-2 vaccine booster^9,26^. Interestingly, we also observed a trend for decreased IFN-γ+IL-2+ and TNF+IL-2+CD4+T cells, which we found to be independently associated with post-vaccination anti-Spike IgG/M/A levels. This, therefore, links decreased T cell immunity with age to decreased humoral immunity.

Due to the inflammatory nature of obesity^27^, and the detrimental role that inflammation plays in antigen-specific immunity^24^, and the data which shows that obesity in humans and mice increases COVID-19 disease severity and increased hospitalisation in humans despite vaccination^28,29^—the impact of obesity on vaccine immunogenicity was investigated. Unlike in the large COVIDENCE cohort where we found increased BMI associated with higher anti-Spike IgG/A/M antibody ratio titres^15^ we did not observe the same relationship in this sub-study. Interesting, despite not identifying any relationship to increased BMI with humoral immunity, BMI category was associated with SARS-CoV-2-specific TNF+CD8+ and CD4+T cells, with TNF+CD8+ T cells independently highly correlated with SARS-CoV-2 neutralising antibody titres. It may be that a non-linear relationship exists between BMI category and neutralising antibodies. We also may not have power to detect this relationship, given we also did not identify the relationship between BMI and anti-Spike IgG/A/M antibody ratios identified in the larger cohort which included more individuals with BMI > 30 (11.8% here vs 18.5% in larger cohort). However, other studies have also either not observed any effect of increased BMI on SARS-CoV-2 vaccination using BNT162b2^10,12^ or showed reduced antibody response post- BNT162b2 or CoronaVac vaccination in individuals with high BMI^30^. The difference between these studies may be due to the lower average BMI observed in our study as compared to previous studies^10,12^.

The main strength of this study was that it was a population-based study in which we applied multivariate analyses to identify independent and associated predictors of immune correlates post-COVID-19 vaccination. In addition, most studies to-date have focussed on BNT162b2 vaccine immunogenicity, the strength of our study is that we assessed vaccine Pfizer BNT162b2 and ChAdOx1-nCoV-19 vaccination allowing us to compare immune responses to the two vaccines administered at the same stage of the pandemic. One finding from our study was that we observed that the concentration of anti-Spike IgG/A/M antibody ratio significantly correlated with antigen-specific CD4+T cell immunity and induced cytokine secretion—demonstrating the strengths of combining a comprehensive characterisation of humoral responses with PBMC and whole blood antigen stimulation assays. The use of the combined IgG/A/M ELISA is another strength of this study, as a previous study has shown that when measuring individual IgG, IgA, and IgM isotypes during the pandemic, it was found that testing the individual isotypes alone was not as sensitive for picking up mild SARS-CoV-2 infections as the combined approach of IgG/A/M—demonstrating that the combined ELISA approach is best for identifying the breath of the antibody response. Another strength of the study was the robust co-variate adjustment ensuring that the differences we observed where due to the variable. The limitations to this study were that we had a bias towards older individuals in the study population, with limited number of people under 35 years old. In addition, there was a modest bias towards female sex (59% vs 41%) in our study population. Another limitation to the study was that it was a single time-point after COVID-19 vaccination, this means we were not able to look at waning immunity over time. It would also have been additive to the study to have collected PBMC samples pre- and post-vaccination, however this was not possible to the fast pace of the pandemic.

To conclude age, vaccine type and prior infection influence the humoral and cellular immune response to SARS-CoV-2 vaccination, whilst BMI affected cellular immunogenicity that independently associated with neutralising antibody levels. We found no difference in vaccine immunogenicity by sex. This study supports the need to include diverse populations during early vaccine efficacy testing, with demographic features related to poor infection outcome, and the benefits of parallel measurement of humoral and cellular immune functions to enable a thorough understanding of vaccine-induced immune correlates of protection.

## Methods

### Study design

This study was a sub-study nested within the CORONAVIT randomised controlled trial as reported elsewhere^16,31^. 6200 U.K. residents aged 16 years or older participated in the COVIDENCE U.K. study as described previously^16^, all of whom provided dried blood spot samples (prior to vaccination) for the determination of combined IgG, IgA and IgM (IgG/A/M) antibody responses to the Spike protein of SARS-CoV-2, as described below. A subset of the participants (123 individuals in total) returned after full informed written consent was obtained, after COVID-19 vaccination and provided a dried blood spot for antibody analysis and a sodium heparin blood sample to assess cellular immunity to SARS-CoV-2 using antigen-stimulated whole blood and peripheral blood mononuclear cell (PBMC) assays. Serum was also collected for C Reactive protein (CRP) and SARS-CoV-2 neutralising antibody quantification. The trial was sponsored by Queen Mary University of London, approved by the Queens Square Research Ethics Committee, London, U.K. (ref 20/HRA/5095), and registered with ClinicalTrials.gov (NCT04579640) on 8 October 2020, before enrolment of the first participant on 28 October 2020.

### In vitro cellular immune response assays

Heparinised blood collected from all sub-study participants was used to assess cellular immunity post vaccination using two approaches:

Whole blood was stimulated in the presence or absence of PepTivator® SARS-CoV-2 Prot_S Complete (1 µg/mL; Miltenyi Biotec) or E. coli lipopolysaccharide (LPS, 1–1000 ng/mL; Invivogen) for 24 h at 37 °C in 5% CO_2_. Following incubation plasma was collected and stored at −80 °C for cytokine assessment by cytometric bead array as detailed below.

PBMCs were isolated from heparinised blood using Ficoll (Merck Life Science) density gradient, washed twice in Hanks Balanced Salt Solution (Merck Life Science) and cryopreserved in 10% dimethylsulphoxide (DMSO) in Fetal Calf Serum (Invitrogen). Subsequently, 5 × 10^5^ cells/condition of cryopreserved PBMCs were recovered and stimulated with nothing (negative control) or PepTivator® SARS-CoV-2 Prot_S Complete (Miltenyi Biotec, 1 µg/mL) or 1 µg/mL of soluble CD3 monoclonal antibody (OKT3, Functional Grade, Invitrogen) for 1 h at 37 °C in 5% CO_2_. Brefeldin A (2.5 µg/mL) was then added to the cells, which were incubated for a further 15 h at 37 °C in 5% CO_2_. Cells were collected and analysed by flow cytometry as detailed below.

### Flow cytometric analysis

Cells were cell surface stained for CD3 (clone: HIT3a; 5 in 100 dilution; catalogue no.300306), CD4 (RPA-T; 3 in 100 dilution; catalogue no.4300528), CD8 (SK1; 2 in 100 dilution; catalogue no. 344732), CD27 (O232; 2 in 100 dilution; catlog no.302832), CD45RA (HI100; 4 in 100 dilution; catlog no.304108) and Zombie NIR viability dye (Biolegend, San Diego, CA, USA) in the presence of Brilliant Buffer (BD Biosciences). Cells were washed and then fixed in Intracellular Fixation Buffer (eBioscience), permeabilised in eBioscience Permeablization Buffer and stained for intracellular IL-2 (JES6-5H4; 1 in 100 dilution; catalogue no. 503810), IFN-γ (4 S.B3; 1 in 100 dilution; catalogue no.502528) and TNF (Mab11, 0.5 in 100 dilution; catalogue no.502932) (Biolegend). Cells were then washed and acquired using the ACEA Novocyte 3000 flow cytometer (Agilent). Data were analysed using FlowJo Version X (BD Biosciences). A representative flow cytometry gating strategy is shown in Supplementary Fig. 1. Following single cell and live cell gating, once CD3+CD4+ and CD8+ cells were identified then proportion cytokine expression was calculated in the stimulated conditions based upon the unstimulated control. In addition, T cell phenotypic staining was performed on unstimulated cells using the markers CD27 and CD45RA to determine if the CD4+ or CD8+ T cell are naive (CD27+CD45RA+), central memory (CM, CD27+CD45RA−), effector memory (EM, CD27-CD45RA-) or senescent-like effector memory re-expressing CD45RA (EMRA; CD27-CD45RA+). A representative flow cytometry gating strategy for T cell memory phenotypes and representative plots from young (<40 years) and old (≥60 years) donors is shown in Supplementary Fig. 2.

### Anti-spike (S) IgG/A/M serology testing

Anti-S IgG/A/M titres were determined by the Clinical Immunology Service at the Institute of Immunology and Immunotherapy of the University of Birmingham using an ELISA that measures combined IgG/A/M responses to the SARS-CoV-2 trimeric Spike glycoprotein (product code MK654, The Binding Site [TBS], Birmingham, U.K.), as previously described^15,32^. This assay has been CE-marked with 98.3% (95% confidence interval [CI] 96.4–99.4) specificity and 98.6% (92.6–100.0) sensitivity for RT-PCR-confirmed mild-to-moderate COVID-19^32,33^, and has been validated as a correlate of protection against breakthrough SARS-CoV-2 infection in two populations^33,34^. A cut-off ratio relative to the TBS assay cut-off calibrators was determined by plotting 624 pre-2019 negatives in a frequency histogram. A cut-off coefficient was then established for IgG/A/M (1.31), with ratio values classed as positive (≥1) or negative (<1). Dried blood spot eluates were pre-diluted 1:40 with 0.05% PBS-Tween using a Dynex Revelation automated absorbance microplate reader (Dynex Technologies). Plates were developed after 10 min using 3,30,5,50-tetramethylbenzidine core, and orthophosphoric acid used as a stop solution (both TBS). Optical densities at 450 nm were measured using the Dynex Revelation.

### SARS-CoV-2 neutralising antibody

Serum titres of neutralising antibodies to SARS-CoV-2 were measured as previously described using an authentic virus (Wuhan Hu-1 strain) microneutralisation assay^35^.

### C-reactive protein assessment

Serum was analysed for C-Reactive protein concentrations using Human C-Reactive Protein/CRP DuoSet ELISA (Biotechne, R+D systems) according to the manufacturers protocol.

### Cytometric bead array

A cytometric bead array to measure IL-8, IL-6, IFN-γ and TNF in whole blood stimulated plasma was carried out according to the manufacturer’s protocol (BD Biosciences). Samples were analysed using the ACEA Novocyte 3000 flow cytometer (Agilent). The lower limit of detection was 1.5 pg/mL.

### Statistical analysis

Principle component analysis (PCA) and general linear modelling (GLM) was conducted using Qlucore Omics Explorer 3.7 (Qlucore AB, Lund, Sweden). Analyte concentrations were log2 converted and normalised to the mean for each analyte with variance -1 to +1. Missing values were imputed by K nearest neighbours (k-NN). Of the 127 participants with acquired data, 12 were excluded from statistical analysis (two due to haematological malignancy with an absence of CD4+T cells, four due to immunodeficiency, three for being on immunosuppressive medication, one as having reported worst health and two who were identified as outliers on PCA (Supplementary Fig. 3). Similarity between two analytes analysed by Pearson correlation. Parameters whose concentration differed significantly between vaccination type, sex and pre-vaccination anti-Spike serostatus were identified using the t-test for GLM, differences based on BMI category, ethnicity and general health category were assessed by multinomial linear regression, whilst parameters affected by age, BMI value and days post second vaccination were identified using a liner regression model and inter-vaccine days were analysed by quadratic regression, all with adjustment for baseline COVIDENCE trial arm allocation [placebo, low vitamin D supplementation, high vitamin D supplementation] although we found no significant effect of vitamin D supplementation on vaccine efficacy in the trial^16^. All baseline and post-vaccination covariates identified in univariate analysis to influence the inflammatory profile, were then reanalysed using the same approach but with additional adjustment for all other identified significant covariates. Covariate adjustment was performed using the eliminated factors approach that fits a multiple regression model to all covariates and subtracts the expression values predicted by this model from the observed values in order to remove covariate effects between patients^36^. Anti-spike ratios pre- and post-vaccination and NAB titres post-vaccination were analysed by quadratic regression, with similar adjustment for all identified baseline and post-vaccination covariates. These analyses yield t-statistics representing the magnitude of difference in concentration of a given parameter between groups being compared (calculated as the regression coefficient for each parameter divided by its standard deviation), *p* values and *q* values (Benjamini–Hochberg false discovery rate. Thresholds of 0.05 were applied for p and q values throughout.

### Reporting summary

Further information on research design is available in the Nature Research Reporting Summary linked to this article.

### Supplementary information

Supplementary Material

REPORTING SUMMARY

## Data Availability

The data that support the findings of this study are available on request from the corresponding author, [ES Chambers]. The data are not publicly available due to it containing information that could compromise the privacy of research participants.
