# Supplementary material for: Influence of individuals’ determinants including vaccine type on cellular and humoral responses to SARS-CoV-2 vaccination
Source: NPJ Vaccines. 2024 May 22;9:87. doi: 10.1038/s41541-024-00878-0 (PMC11111746; doi:10.1038/s41541-024-00878-0)
Supplement: Supplementary file 1 — Supplementary Material [file 41541_2024_878_MOESM1_ESM.pdf]

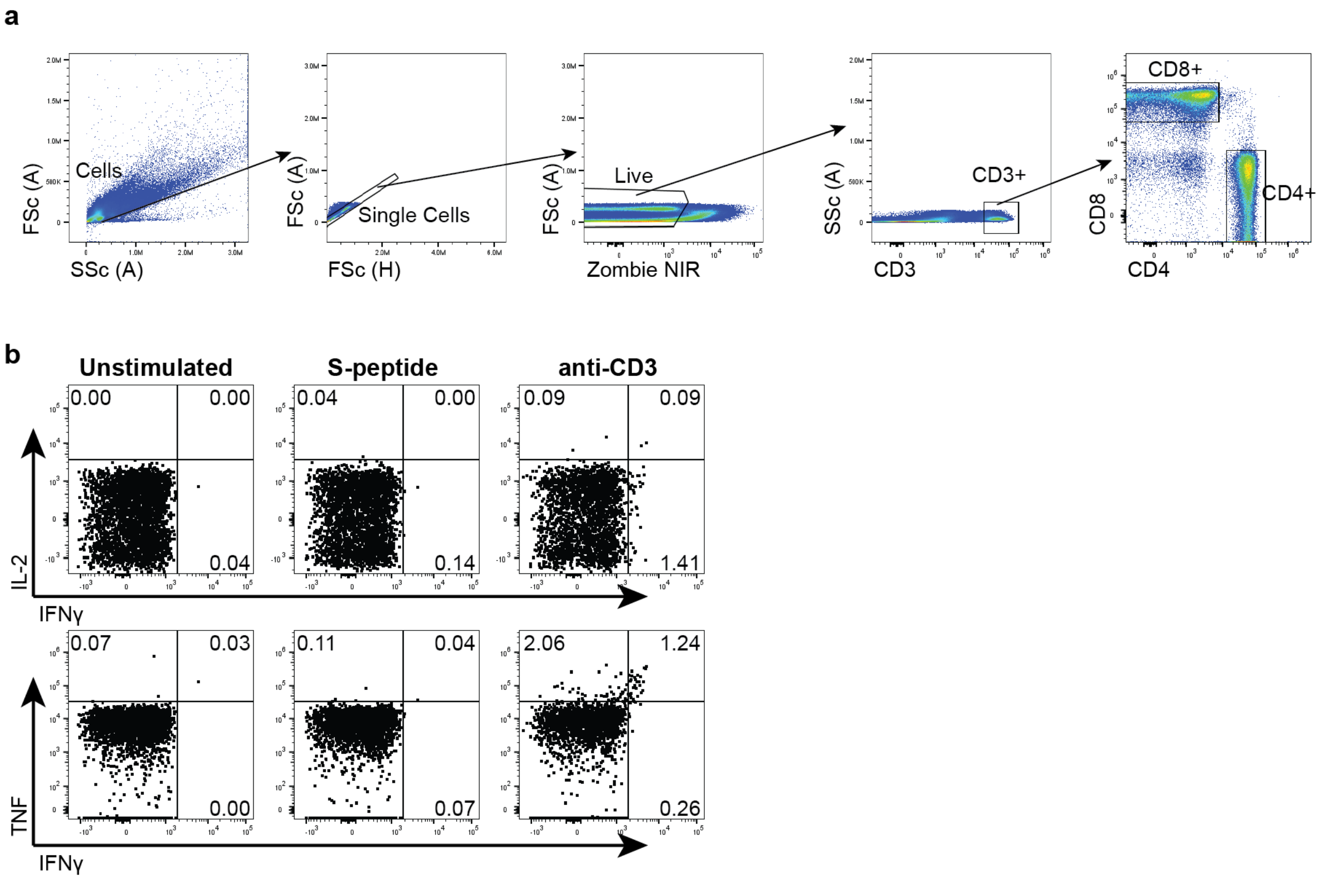

Supplementary Figure 1: Representative flow cytometry gating strategy to identify T cells and intracellular cytokines.

PBMCs were assessed by cell surface and intracellular flow cytometry analysis. **A,** Representative gating strategy to determine CD4+ and CD8+ T cells. Lymphocytes were identified from Forward (FSc) and Side (SSc) scatter plots, subsequently single cells were identified. Then Live cells were selected as being Zombie Near Infrared (NIR) Live dead stain negative, then T cells were identified based upon CD3

expression. Finally CD4+ and CD8+ T cells were identified within the CD3 gate. **B,** Representative IFNy, IL-2 and TNF staining is shown of unstimulated (negative control), S-peptide stimulated and soluble anti-CD3 stimulated (positive control) conditions.

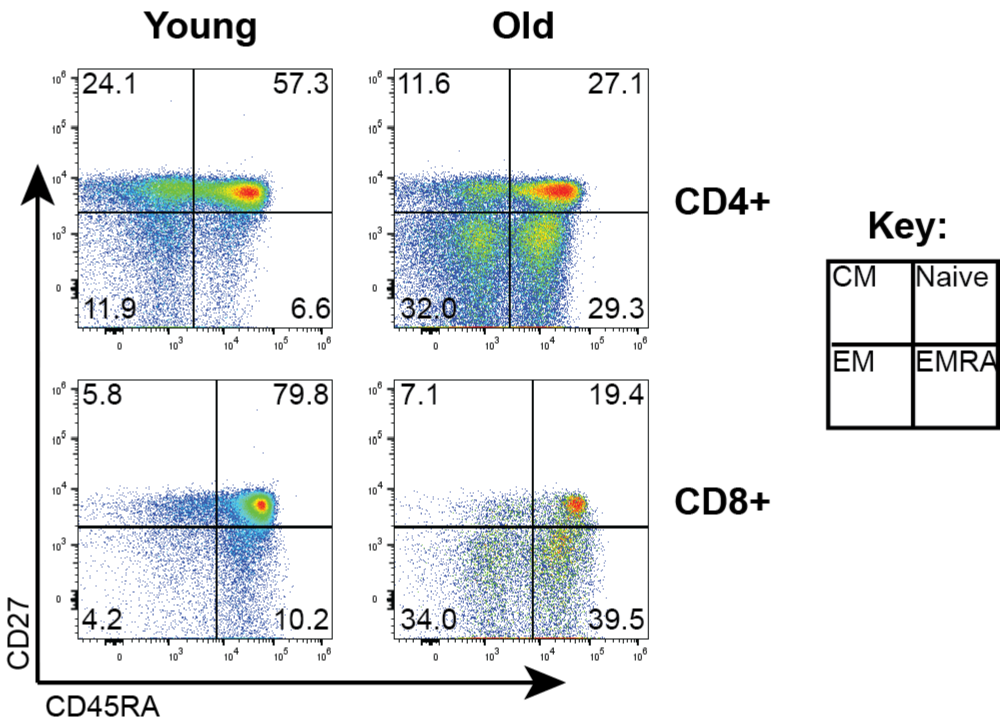

Supplementary Figure 2: Representative gating strategy for phenotyping of T cells.

CD4+ and CD8+ T cells were identified within the CD3 gate, as shown in supplementary Figure 1. Representative CD45RA and CD27 staining shown in CD4+ and CD8+ T cells from a younger (<40 years) and older (≥65 years) person from unstimulated PBMCs. CD45RA+CD27- are considered naïve, CD45RA-CD27+ are considered Central memory (CM), CD45RA-CD27- Effector memory (EM) and CD27-CD45RA+ are senescent-like T cells known as Effector Memory re-expressing CD45RA (EMRA).

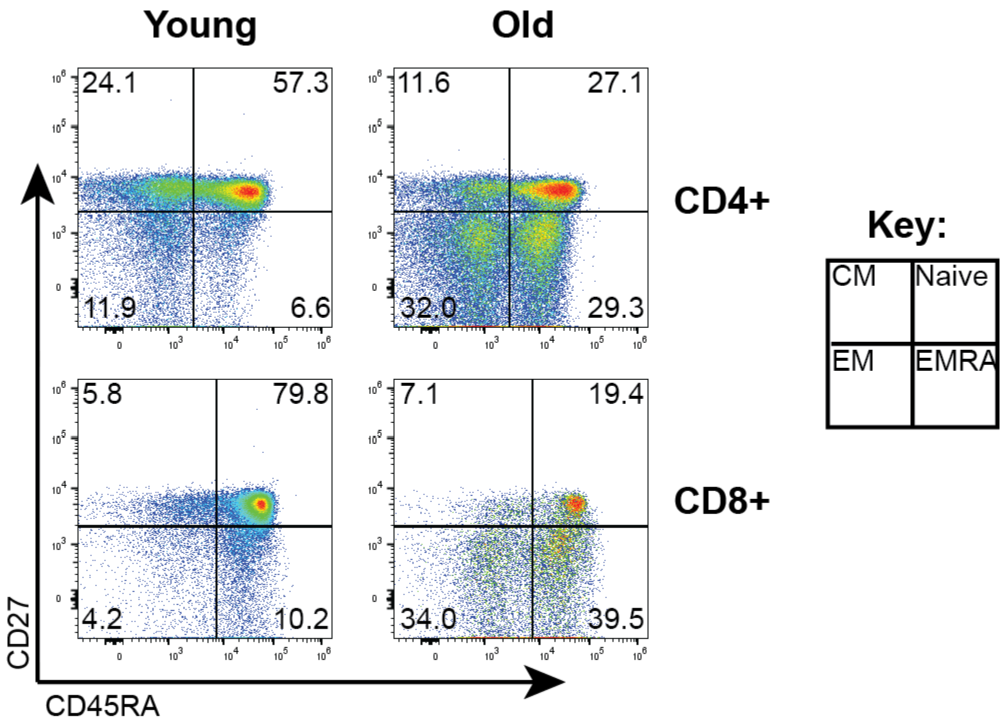

Supplementary Figure 2: Representative gating strategy for phenotyping of T cells.

CD4+ and CD8+ T cells were identified within the CD3 gate, as shown in supplementary Figure 1. Representative CD45RA and CD27 staining shown in CD4+ and CD8+ T cells from a younger (<40 years) and older (≥65 years) person from unstimulated PBMCs. CD45RA+CD27- are considered naïve, CD45RA-CD27+ are considered Central memory (CM), CD45RA-CD27- Effector memory (EM) and CD27-CD45RA+ are senescent-like T cells known as Effector Memory re-expressing CD45RA (EMRA).

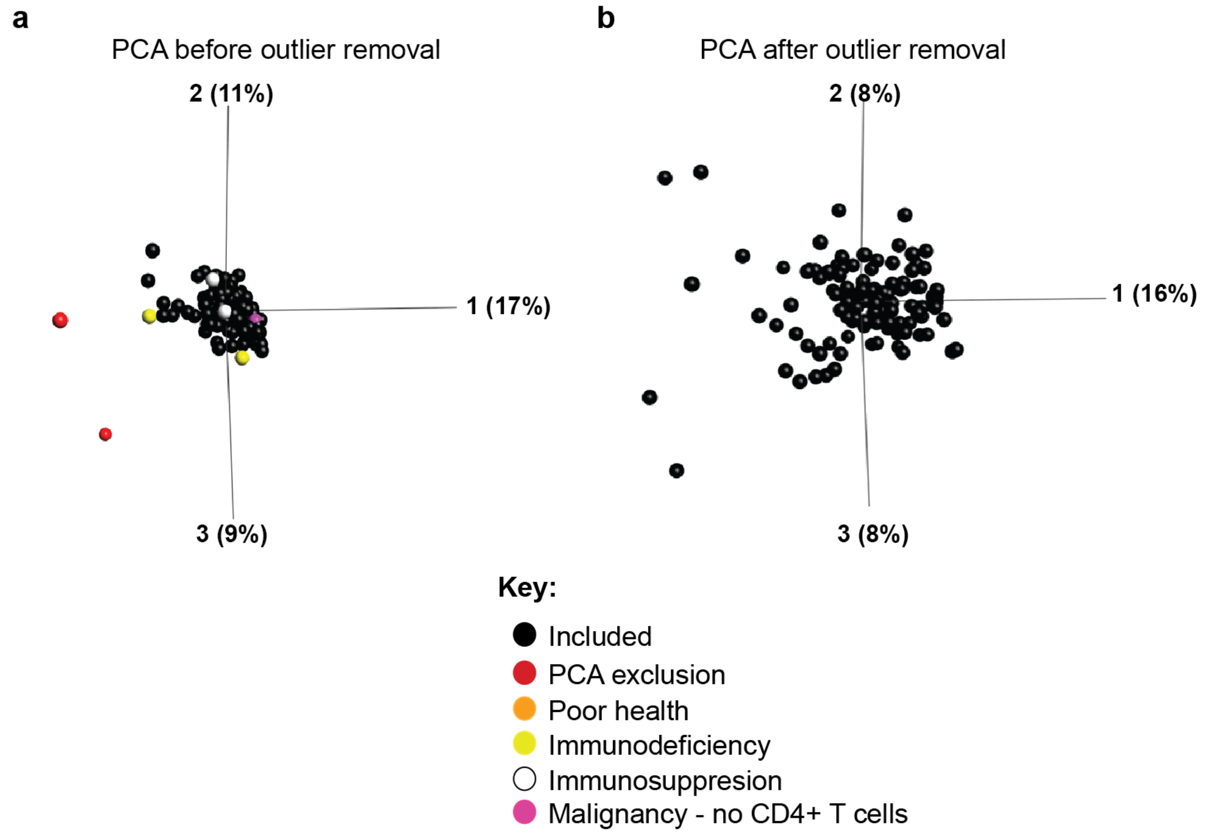

Supplementary Figure 3: Principal component analysis (PCA) before and after outlier removal.

A, Twelve samples were excluded in total. Two samples were identified based upon principle component analysis (PCA) as being outliers and were removed (red). Additionally samples were excluded due to immunosuppressive medication (white; n=3) or immunodeficiency (yellow; n=4) or poorest general health (orange, n=1) or as having malignancy and absence of CD4+ T cells (as determined by flow cytometry; pink; n=2). **B,** PCA plot of all included samples (n=115; black) used for analyses showing good separation of samples across the first three components.

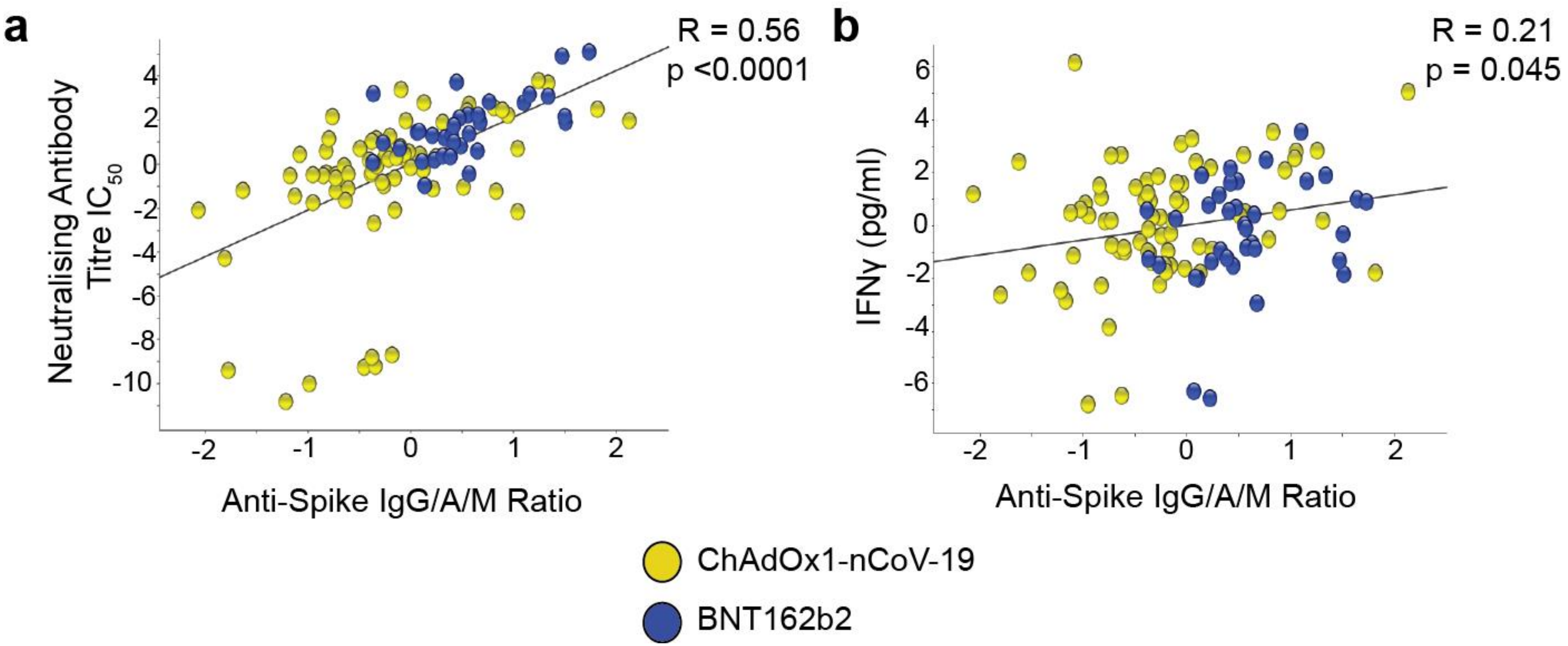

Supplementary Figure 4: Humoral and cellular correlates of anti-S IgG/A/M antibody ratio separated by vaccination received.

Correlation between post-COVID-19 vaccine anti-S IgG/A/M antibody ratio and **A,** neutralising antibody titre IC_{50}, **B,** whole blood IFNγ production after S peptide stimulation coloured according to vaccination received either ChAdOx1-nCoV-19 (yellow) or BNT162b2 (blue). Data presented on x and y axes are normalised including $\log_2$ transformation and adjusted for the baseline and post-vaccination covariates covariates (age, sex, BMI category, pre-vaccine SARS-CoV-2 sero-status, vitamin D randomisation, inter-vaccine days, and days post second

vaccine), p values derived using the quadratic regression for general linear models with adjustment for the same covariates, all $q<0.01$. Trend line indicates Pearson correlation (R-statistic).

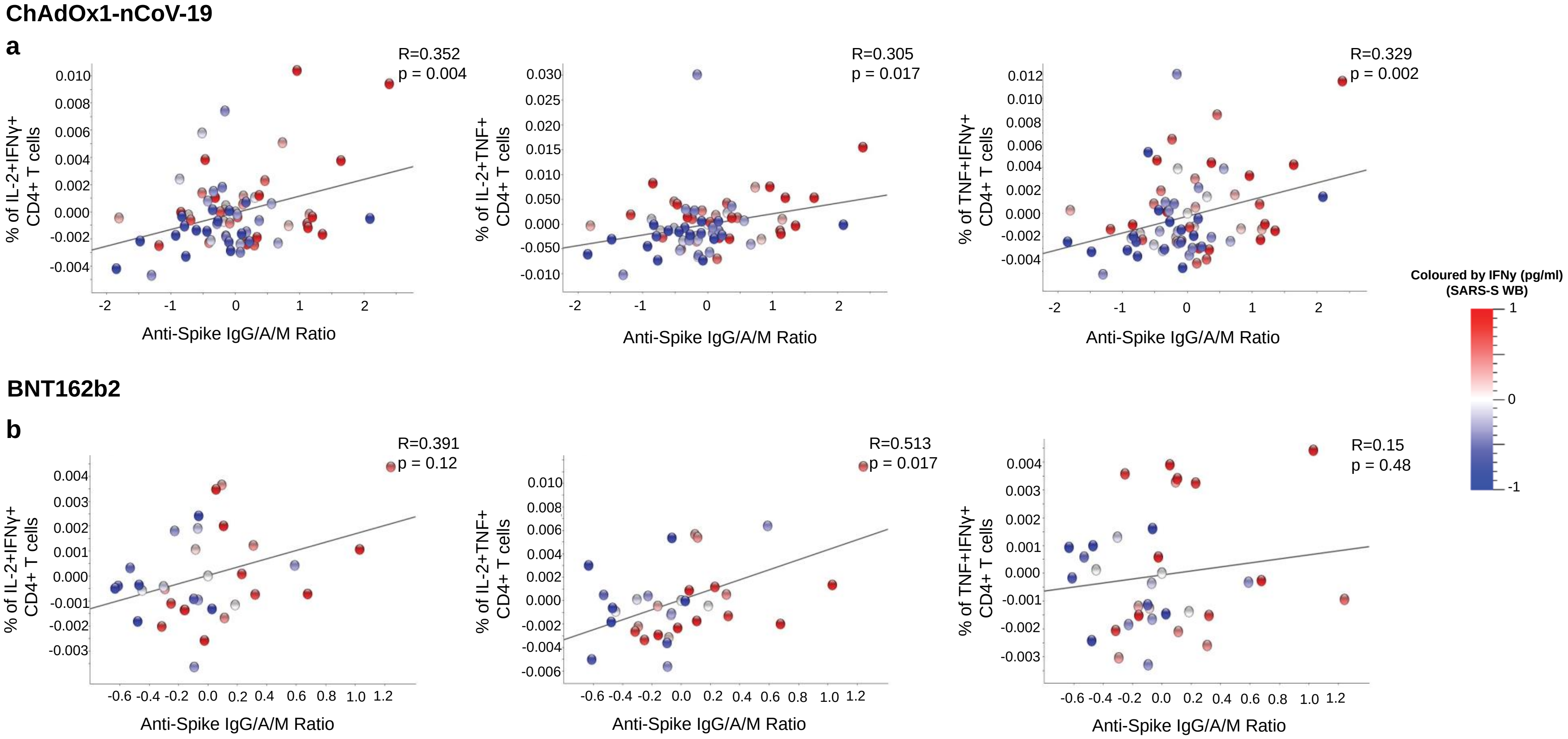

Supplementary Figure 5: Polyfunctional CD4+ T cell correlates of anti-S IgG/A/M antibody ratio after COVID-19 vaccination separated according to vaccination received.

Correlation between post-COVID-19 vaccine anti-S IgG/A/M antibody ratio and percent of cytokine positive CD4+ T after PBMC stimulation with S peptide as determined by intracellular cytokine staining in **A,** individuals who received ChAdOx1-nCoV-19 and **B,** individuals who received

BNT162b2. Coloured according to IFNγ production from S peptide stimulated whole blood (SARS-S WB). Data presented on x and y axes are normalised including log_2 transformation and adjusted for the baseline and post-vaccination covariates (age, sex, BMI category, pre-vaccine SARS-CoV-2 sero-status, vitamin D randomisation, inter-vaccine days, and days post second vaccine), trend line indicates Pearson correlation (R-statistic).

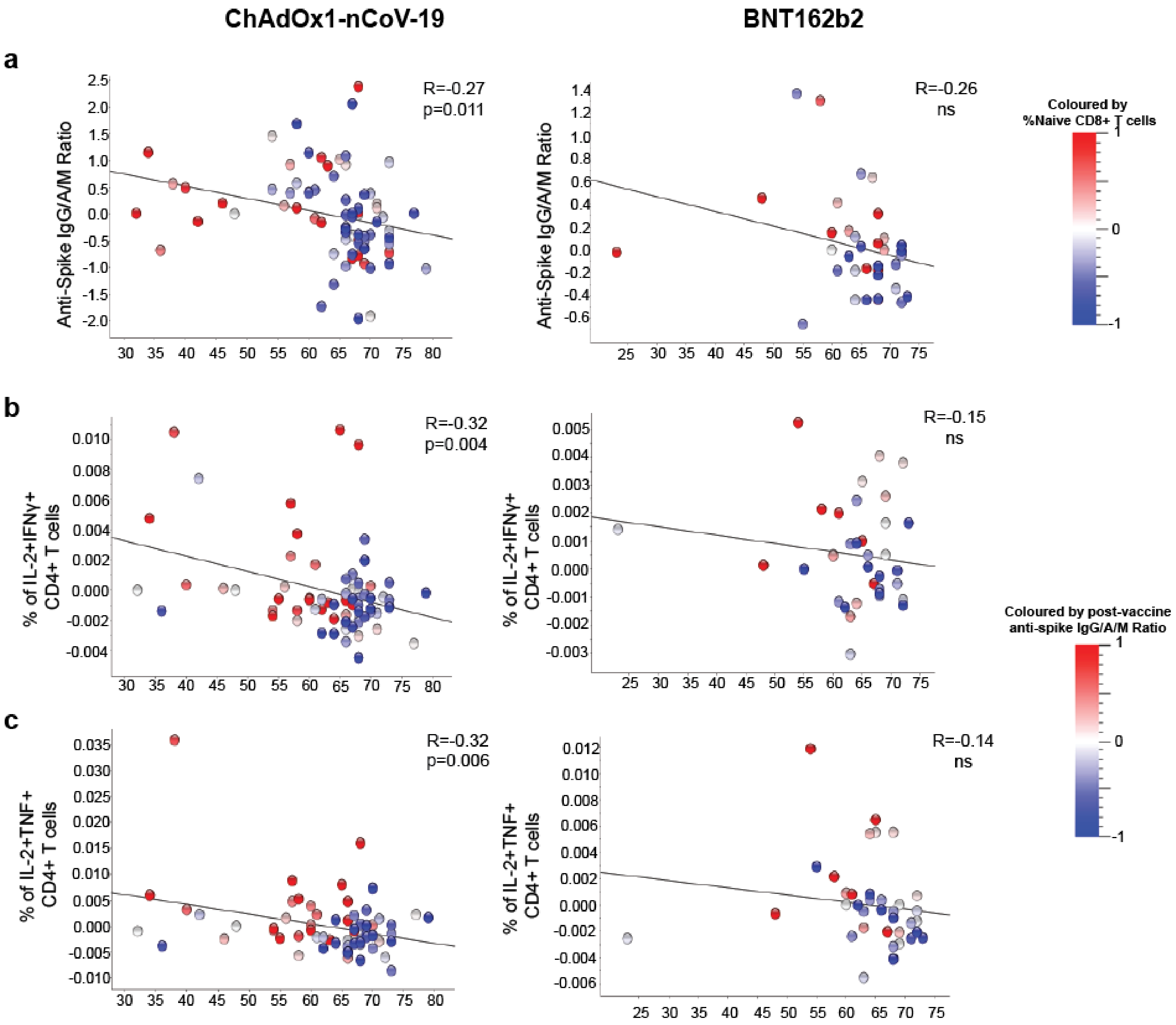

ChAdOx1-nCoV-19
BNT162b2
a
Anti-Spike IgG/A/M Ratio
R=-0.27
p=0.011
R=-0.26
ns
Coloured by
%Naive CD8+ T cells
b
% of IL-2+IFNγ+
CD4+ T cells
R=-0.32
p=0.004
R=-0.15
ns
Coloured by post-vaccine
anti-spike IgG/A/M Ratio
c
% of IL-2+TNF+
CD4+ T cells
R=-0.32
p=0.006
R=-0.14
ns

Supplementary Figure 6: Relationship between post-vaccination anti-S IgG/A/M antibody ratio, percent naïve CD8+ T cells and age separated according to vaccine received.

Correlation between age and **A,** post-vaccine anti-Spike IgG/A/M antibody ratio, coloured according to frequency of naïve (CD45RA+CD27+) CD8+ T cells present in the peripheral blood of the individuals**. B,** percent of IL-2+IFNγ+ cytokine positive CD4+ T after PBMC stimulation with S peptide as determined by intracellular cytokine staining, and **C,** percent of IL-2+TNF+ cytokine positive CD4+ T after PBMC stimulation with S peptide as determined by intracellular cytokine staining, B and C coloured according to anti-S IgG/A/M antibody ratio normalised to mean 0 and variance ±1. Data presented on y-axes is normalised including log2 transformation and adjusted for the covariates baseline and post-vaccination covariates (sex, BMI category, pre-vaccine SARS-CoV-2 sero-status, vitamin D randomisation, inter-vaccine days, and days post second vaccine), p values derived using linear regression for general linear models with adjustment for the same covariates, all $q<0.07$. Trend line indicates Pearson correlation (R-statistic).

		ChAdOx1 nCoV-19 (n=77)	BNT162b2 (n=38)
Age	Median age, years (IQR)	66.4 (60.4–68.9)	66.4 (62.3–68.7)
	Age range, years	31.7–78.6	23.5–73.1
Sex, N (%)	Male	27 (35%)	20 (53%)
	Female	50 (65%)	18 (47%)
Ethnicity, N (%)	White	72 (94%)	35 (92%)
	South Asian	1 (1%)	0
	Black/African/Caribbean/ Black British	0	0
	Mixed/Multiple/Other	4 (5%)	3 (8%)
Body mass index, kg/m^2, N (%)	<25	39 (51%)	18 (47%)
	25-30	29 (38%)	16 (42%)
	>30	9 (12%)	4 (11%)
Highest educational level attained, N (%) [3]	Primary/Secondary	5 (6%)	4 (11%)
	Higher/Further (A levels)	8 (10%)	1 (3%)
	College	39 (51%)	16 (42%)
	Post-graduate	25 (32%)	17 (45%)
Quantiles of IMD decile, N (%)	Q1 (most deprived)	14 (18%)	5 (13%)
	Q2	12 (16%)	9 (24%)
	Q3	24 (31%)	14 (37%)
	Q4 (least deprived)	27 (35%)	10 (26%)
Tobacco smoking, N (%)	Non-current/never smoker	73 (95%)	38 (100%)
	Current smoker	4 (5%)	0
Alcohol consumption/week, units, N (%)	None	13 (17%)	8 (21%)
	1-7	30 (39%)	13 (34%)
	8-14	19 (25%)	7 (18%)
	15-21	9 (12%)	7 (18%)
	22-28	5 (6%)	3 (8%)
	>28	1 (1%)	0 (0%)
Self-assessed general health, N (%)	Excellent	20 (26%)	9 (24%)
	Very good	28 (36%)	15 (41%)
	Good	22 (29%)	9 (24%)
	Fair	7 (9%)	4 (11%)
	Poor	0	0
Pre-vaccination anti-spike IgG/A/M serostatus, N (%)	Negative	61 (79%)	33 (87%)
	Positive	11 (14%)	3 (8%)
	Unknown	5 (6%)	2 (5%)
Post-vaccination anti-spike IgG/A/M serostatus, N (%)	Negative	67 (88%)	32 (89%)
	Positive	9 (12%)	4 (11%)
Median inter-dose interval, weeks (IQR)		11.0 (10.0–11.3)	10.7 (9.3–11.0)

Median time from date of second vaccine dose to date of sampling, weeks (IQR)		11.8 (10.1–14.6)	12.2 (9.2–15.0)

Supplementary Table 1: Individual Characteristics split according to whether they received ChAdOx1 nCoV-19 (n=77) or BNT162b2 (n=38).

Abbreviations: IQR, inter-quartile range; s.d., standard deviation; Ig, Immunoglobulin. Self-assessed general health is in 115 participants, and median time from date of second vaccine was measured in 112 participants.

Analyte	t-statistic	p-value	q-value
**CD8% naive**	**-8.233596**	**5.67E-13**	**2.67E-11**
**CD8% EM**	**3.9042053**	**0.0001682**	**0.00395254**
**anti-S**	**-3.257059**	**0.0015206**	**0.02382234**
**CD8% CM**	**3.0910258**	**0.00256**	**0.02525635**
**CD8% EMRA**	**3.0753038**	**0.0026868**	**0.02525635**
**CD4% SARS-S IL-2 IFNg**	**-2.686765**	**0.0084011**	**0.06580853**
**CD4% naive**	**-2.384775**	**0.0189009**	**0.08075836**
**CD4% SARS-S TNF IL-2**	**-2.413737**	**0.017538**	**0.08075836**
**CD8% CD3stim IL-2**	**2.4477711**	**0.0160486**	**0.08075836**
**CD8% CD3stim TNF**	**2.4700887**	**0.0151343**	**0.08075836**
**CD8% CD3stim TNF IL-2**	**2.4610097**	**0.0155005**	**0.08075836**
**CD4% EM**	**2.1953783**	**0.0303593**	**0.11890727**
**CD4% CM**	**2.0655732**	**0.041355**	**0.14951413**
Neut Ab	-1.73968	0.0848736	0.28493278

Supplementary Table 2a. Age univariate analysis. t-statistic (regression co-efficient / standard deviation) represents magnitude of relationship between each immunological parameter to year of age. p values and q values (false discovery rate) derived using linear regression for general linear models with adjustment for vitamin D allocation

Analyte	t-statistic	p-value	q-value
**LPS IL-6**	**4.0386643**	**0.0001031**	**0.004844**
**CD8% EM**	**3.7296636**	**0.0003126**	**0.0073452**
**CD4% EM**	**3.1211045**	**0.0023327**	**0.0280662**
**CD8% naive**	**-3.113463**	**0.0023886**	**0.0280662**
**CD4% CM**	**2.5903513**	**0.0109629**	**0.1030512**
**CD4 naive%**	**-2.401535**	**0.0181012**	**0.1417927**
WB LPS IL-8	2.2255459	0.0282031	0.1893638
CD4% CD3stim TNF IFNg	2.0116978	0.0468381	0.2578707
CD8 CM%	1.9885378	0.0493795	0.2578707
CD4% CD3stim IFNg	1.9021915	0.0599126	0.2815894
WB SARS-S TNF	1.8073611	0.0735969	0.3144597

Supplementary Table 2b. Sex univariate analysis. t-statistic (regression co-efficient / standard deviation) represents magnitude of difference between sexes; a positive t-statistic indicates a higher value and negative a lower value of immunological parameter in males participants compared to female. p values and q values (false

discovery rate) derived using the t-test for general linear models with adjustment for vitamin D allocation.

Analyte	F-statistic	p-value	q-value
CD4% CD3stim IL-2 IFNg	4.5022945	0.0133497	0.3137187
CD4% SARS-S IL-2 IFNg	4.5649233	0.012603	0.3137187
CD4% SARS-S IFNg	3.7444417	0.0269284	0.3515418
WB IL8	3.393244	0.0373981	0.3515418
anti-S	3.6297765	0.0299696	0.3515418

Supplementary Table 2c. Ethnicity univariate analysis. F-statistic (mean sum of squares regression / mean sum of squares error) represents the degree of relatedness of each immunological parameter to Ethnicity (White, Asian, Mixed/Other) category. p values and q values (false discovery rate) derived using ANOVA for general linear models with adjustment for vitamin D allocation.

Analyte	F-statistic	p-value	q-value
anti-S	2.0696485	0.09031685	0.7748185
CD4% naive	2.0019493	0.09991301	0.7748185

Supplementary Table 2d. General Heath category univariate analysis. logical parameter to General Health category (0-4). p values and q values (false discovery rate) derived using ANOVA for general linear models with adjustment for vitamin D allocation.

Analyte	t-statistic	p-value	q-value
**Neut Ab**	**-3.917003**	**0.0001606**	**0.00377425**
**anti-S**	**-4.029426**	**0.0001066**	**0.00377425**
CD4% SARS-S IL-2	2.2333531	0.0276674	0.43345593
WB LPS TNF	-1.906888	0.0592946	0.69671118

Supplementary Table 2e. Vaccine Type univariate analysis. t-statistic (regression co-efficient / standard deviation) represents magnitude of difference between vaccine types; a positive t-statistic indicates a higher value and negative a lower value of immunological parameter in participants receiving BNT162b2 as compared to ChAdOx1-nCoV-19. p values and q values (false discovery rate) derived using the t-test for general linear models with adjustment for vitamin D allocation.

Analyte	F-statistic	p-value	q-value
**CD4% SARS-S TNF IFNg**	**6.1162329**	**0.0030904**	**0.1292561**
**CD8% SARS-S TNF**	**5.4748621**	**0.0055003**	**0.1292561**
postvax_25D	4.2226462	0.0172758	0.2706541
CD4% SARS-S IL-2 IFNg	3.554971	0.0321403	0.3776491
CD8% CD3stim TNF	3.1552916	0.0467749	0.4396844
CD4% EMRA	2.8732269	0.0610575	0.478284

Supplementary Table 2f. Inter-vaccine Days univariate analysis. F-statistic (mean sum of squares regression / mean sum of squares error) represents the degree of relatedness of each immunological parameter to the number of inter-vaccine days. p values and q values (false discovery rate) derived using the quadratic regression for general linear models with adjustment for vitamin D allocation.

Analyte	F-statistic	p-value	q-value
**WB SARS-S IL-6**	**4.9951754**	**2.38E-06**	**0.0001119**
**WB SARS-S IL-8**	**4.542202**	**1.50E-05**	**0.0003535**
**WB SARS-S TNF**	**3.222728**	**0.0016961**	**0.0265722**
WB TNFa	2.4030347	0.0180311	0.2118654
postvax_25D	2.2852092	0.0243299	0.2287008
WB LPS IFNg	1.9209512	0.057476	0.3859105
Neut Ab	-1.969867	0.0515128	0.3859105
CD8% CD3stim IL-2 IFNg	-1.663729	0.099177	0.4456425
CD8% SARS-S TNF	1.7252017	0.0874615	0.4456425

Supplementary Table 2g. Days post second vaccine univariate anlaysis. F-statistic (mean sum of squares regression / mean sum of squares error) represents the degree of relatedness of each immunological parameter to the number of days after the second vaccine and blood sampling. p values and q values (false discovery rate) derived using the quadratic regression for general linear models with adjustment for vitamin D allocation.

Analyte	t-statistic	p-value	q-value
**anti-S**	**-3.936033**	**0.00014993**	**0.00704652**
**CD4% SARS-S IFNg**	**-3.607106**	**0.00047759**	**0.01122342**
WB SARS-S IFNg	-2.399104	0.01821529	0.28537288
WB LPS IL-8	2.1356061	0.03505882	0.41194117

Supplementary Table 2h. Pre-vaccination seropositivity univariate analysis. t-statistic (regression co-efficient / standard deviation) represents magnitude of difference between those SARS-CoV-2 seropositive pre-vaccination to those sero negative. p values and q values (false discovery rate) derived using the t-test for general linear models with adjustment for vitamin D allocation.

Analyte	F-statistic	p-value	q-value
**CD4% SARS-S TNF**	**13.23766**	**2.29E-07**	**1.08E-05**
**CD8% SARS-S TNF**	**9.4983377**	**1.37E-05**	**0.00032184**
**CD4% SARS-S IL-2**	**3.6947098**	**0.0142727**	**0.13854697**
**CRP**	**3.6767092**	**0.0145975**	**0.13854697**
**CD4% EM**	**3.6689937**	**0.014739**	**0.13854697**
CD8% EMRA	2.8795691	0.0396169	0.31033205
CD8% CD3stim IFNg	2.4184527	0.0705411	0.35958989
CD8% CM	2.3780999	0.0741847	0.35958989
CD8% CD3stim TNF IFNg	2.3545861	0.076393	0.35958989
WB LPS IL-6	2.3533747	0.0765085	0.35958989
CD8% CD3stim TNF	2.2703345	0.0848514	0.36254674
CD8% SARS-S IL-2	2.1643517	0.0968113	0.37917779

Supplementary Table 2i. BMI category univariate analysis. F-statistic (mean sum of squares regression / mean sum of squares error) represents the degree of relatedness of each immunological parameter to BMI category (1-4). p values and q values (false discovery rate) derived using ANOVA for general linear models with adjustment for vitamin D allocation.

Analyte	t-statistic	p-value	q-value
**CRP**	**3.6066625**	**0.0004783**	**0.0224809**
CD8% CD3stim IL-2	2.0970855	0.0384108	0.8127592
CD8% CM	1.8509272	0.0670179	0.8127592
CD8% SARS-S IL-2	-1.811537	0.072944	0.8127592

Supplementary Table 2i. BMI value univariate analysis. t-statistic (regression co-efficient / standard deviation) represents magnitude of relationship between each immunological parameter to BMI value. p values and q values (false discovery rate) derived using linear regression for general linear models with adjustment for vitamin D allocation.

Supplementary Tables 2a-i: Factors with analytes significant at q-value <0.15 on unadjusted univariate analysis were included as covariates for adjustment in subsequent analyses.

BMI category was selected over BMI value. Neut Ab = post-vaccine neutralising antibody; SARS-S = Stimulated with SARS-CoV-2 peptide; CD4% = percent of CD4+ T cells; CD8% = percent of CD8+ T cells; CM = central memory; EM = effector memory; EMRA = Effector memory re-expressing CD45RA; WB = whole blood stimulation assay; CRP = C Reactive protein; LPS = lipopolysaccharide stimulated; CD3stim= PBMCs stimulated with anti-CD3.

Analyte	F-statistic	p-value	q-value
**Neut Ab**	**14.75143623**	**0.000002510**	**0.000112931**
**CD4% SARS-S IL-2 IFNy**	**8.903148651**	**0.000280028**	**0.006300625**
**CD4% SARS-S TNF IL-2**	**8.350981712**	**0.000447823**	**0.006717338**
**CD4% SARS-S TNF IFNy**	**7.354140282**	**0.001057407**	**0.009634061**
**WB SARS-S IFNy**	**7.340041637**	**0.001070451**	**0.009634061**
CD4% SARS-S IL-2	4.408493519	0.014679182	0.110093862
CD4% CM	0.661622703	0.518302761	0.915459568
CD4% EM	0.43418476	0.649032549	0.915459568
CD4% EMRA	0.97393471	0.381218715	0.915459568
CD4% CD3stim IFNy	0.533860683	0.588032301	0.915459568
CD4% CD3stim IL-2 IFNy	1.042005539	0.356621314	0.915459568
CD4% CD3stim TNF IFNy	0.610551715	0.545103709	0.915459568
CD4% CD3stim TNF IL-2	0.35597381	0.701392942	0.915459568
CD4% SARS-S TNF	0.340823352	0.712024108	0.915459568
CD8% CM	1.465167642	0.23605523	0.915459568
CD8% EM	0.35681048	0.700810586	0.915459568
CD8% EMRA	1.355161548	0.262695259	0.915459568
CD8% CD3stim IFNy	1.322524309	0.271174859	0.915459568
CD8% CD3stim IL-2	1.122089267	0.329745445	0.915459568
CD8% CD3stim TNF	0.590034723	0.556264973	0.915459568
CD8% CD3stim TNF IFNy	1.095577955	0.338405755	0.915459568
CD8% CD3stim TNF IL-2	0.942824781	0.393030177	0.915459568
CD8% SARS-S IFNy	0.99430573	0.37368131	0.915459568
CD8% SARS-S IL-2 IFNy	0.347255528	0.70749067	0.915459568
CD8% SARS-S TNF	0.695522726	0.501258821	0.915459568
CD8% SARS-S TNF IFNy	0.579966128	0.561827329	0.915459568
CD8% SARS-S TNF IL-2	1.058355212	0.350958456	0.915459568
CRP	0.408156067	0.666000048	0.915459568
WB LPS IFNy	0.659529507	0.519374345	0.915459568
WB LPS IL-6	0.411960125	0.663492304	0.915459568
WB LPS IL-8	1.36215663	0.260913333	0.915459568
WB SARS-S IL-6	1.501778722	0.227814238	0.915459568
WB SARS-S IL-8	1.356493115	0.262355101	0.915459568
WB SARS-S TNF	0.866638422	0.423558472	0.915459568
WB TNF	0.485486239	0.616870022	0.915459568
CD4% naive	0.311279863	0.733230398	0.916537997
CD4% CD3stim TNF	0.217790619	0.804682057	0.928479296
CD8% SARS-S IL-2	0.242988914	0.784751311	0.928479296
WB IL6	0.237713635	0.788881718	0.928479296
CD4% SARS-S IFNy	0.177802429	0.837377229	0.942049383
CD4% CD3stim IL-2	0.041483454	0.959382046	0.959400468

CD8% naive	0.051724799	0.949616066	0.959400468
CD8% CD3stim IL-2 IFNy	0.122665226	0.884695338	0.959400468
WB LPS TNF	0.079138882	0.923970584	0.959400468
WB IL8	0.041464236	0.959400468	0.959400468

Supplementary Table 3: Immune correlates of post-vaccine Anti-S IgG/A/M antibody ratio adjusting for baseline and post-vaccination covariates

F-statistic (mean sum of squares regression / mean sum of squares error) represents the degree of relatedness of each immunological parameter to post-vaccine anti-S IgG/A/M antibody levels. p values and q values (false discovery rate) derived using the quadratic regression for general linear models with adjustment for the following covariates: age, sex, BMI category, vaccine types, inter-vaccine days, days post second vaccine, pre-vaccine SARS-CoV-2 sero-status, vitamin D allocation.

Neut Ab = post-vaccine neutralising antibody; SARS-S = Stimulated with SARS-CoV-2 peptide; CD4% = percent of CD4+ T cells; CD8% = percent of CD8+ T cells; CM = central memory; EM = effector memory; EMRA = Effector memory re-expressing CD45RA; WB = whole blood stimulation assay; CRP = C Reactive protein; LPS = lipopolysaccharide stimulated; CD3stim= PBMCs stimulated with anti-CD3.

Analyte	F-statistic	p-value	q-value
**anti-S**	**18.82341003**	**1.37E-07**	**6.15E-06**
**CD8% SARS-S TNF**	**9.043599129**	**0.00025768**	**0.005797797**
WB LPS IFNg	4.476495266	0.013927031	0.208905463
CD4% SARS-S IL-2 IFNg	4.155908108	0.018675077	0.210094621
CD4% SARS-S TNF IFNg	3.773694038	0.026559214	0.239032924
CD4% SARS-S TNF IL-2	2.733723402	0.070201699	0.478347739
CD8% CD3stim TNF	2.672126532	0.074409648	0.478347739
CD8% SARS-S IFNg	2.120285988	0.125761624	0.707409137
CD8% naive	1.922573924	0.151997455	0.759987273
WB LPS IL-6	1.616731644	0.204076375	0.865460951
WB LPS IL-8	1.579493642	0.211557121	0.865460951
CD8% CD3stim TNF IL-2	1.480389357	0.232861184	0.873229441
CD8% CD3stim IL-2	1.285854697	0.281282137	0.9144346
CD8% SARS-S TNF IFNg	1.2453866	0.292584657	0.9144346
CRP	1.203369021	0.304811533	0.9144346
CD4% EMRA	0.43726778	0.647119287	0.979267193
CD4% CD3stim IFNg	0.671686947	0.513306603	0.979267193
CD4% CD3stim IL-2	0.423572212	0.655960522	0.979267193
CD4% CD3stim TNF	0.556580186	0.575063077	0.979267193
CD4% CD3stim TNF IFNg	0.639137626	0.530049424	0.979267193
CD4% CD3stim TNF IL-2	0.743589699	0.478209243	0.979267193
CD4% SARS-S IFNg	0.705029428	0.496715404	0.979267193
CD8% EM	0.514346242	0.59958208	0.979267193
CD8% EMRA	0.940470517	0.39412536	0.979267193
CD8% CD3stim IL-2 IFNg	0.792571187	0.455712813	0.979267193
CD8% CD3stim TNF IFNg	0.395296782	0.674606289	0.979267193
CD8% SARS-S TNF IL-2	0.733572781	0.482947855	0.979267193
WB LPS TNF	0.879765749	0.418304014	0.979267193
WB SARS-S IFNg	0.863521218	0.425027598	0.979267193
WB SARS-S IL-8	0.406051397	0.667451352	0.979267193
WB SARS-S TNF	0.497693449	0.609541115	0.979267193
CD4% EM	0.170627251	0.843399134	0.998762132
CD4% naive	0.224338278	0.799475929	0.998762132
CD4% SARS-S IL-2	0.171836704	0.842383437	0.998762132
CD4% SARS-S TNF	0.274259329	0.76074756	0.998762132
CD8% CD3stim IFNg	0.256668478	0.774170844	0.998762132
CD8% SARS-S IL-2	0.221605882	0.801652948	0.998762132
WB SARS-S IL-6	0.262439609	0.769740529	0.998762132
CD4% CM	0.075825788	0.927034931	0.999529529
CD4% CD3stim IL-2 IFNg	0.03582282	0.96482453	0.999529529
CD8% CM	0.109211959	0.896655177	0.999529529

CD8% SARS-S IL-2 IFNg	0.045929633	0.955130819	0.999529529
WB IL6	0.004542075	0.995468446	0.999529529
WB IL8	0.063582912	0.938437068	0.999529529
WB TNF	0.000470584	0.999529529	0.999529529

Supplementary Table 4: Immune correlates of post-vaccine neutralising antibody titre adjusting for baseline and post-vaccination covariates

F-statistic (mean sum of squares regression / mean sum of squares error) represents the degree of relatedness of each immunological parameter to post-vaccine neutralising antibody titre. p values and q values (false discovery rate) derived using the quadratic regression for general linear models with adjustment for the following covariates: age, sex, BMI category, vaccine types, inter-vaccine days, days post second vaccine, pre-vaccine SARS-CoV-2 sero-status, vitamin D allocation.

anti-S = anti-S IgG/A/M antibody ratio; SARS-S = Stimulated with SARS-CoV-2 peptide; CD4% = percent of CD4+ T cells; CD8% = percent of CD8+ T cells; CM = central memory; EM = effector memory; EMRA = Effector memory re-expressing CD45RA; WB = whole blood stimulation assay; CRP = C Reactive protein; LPS = lipopolysaccharide stimulated; CD3stim= PBMCs stimulated with anti-CD3.

**Analyte**	**F-statistic**	**p-value**	**q-value**
**CD4% SARS-S IFNg**	**27.42686631**	**2.82E-10**	**1.30E-08**
**anti-S**	**14.86001601**	**2.14E-06**	**4.92E-05**
**CD8% SARS-S IFNg**	**8.333600224**	**0.000441975**	**0.006776952**
**CD4 EM%**	**5.302874986**	**0.006426891**	**0.073909249**
CD4 EMRA%	3.246207551	0.042937748	0.386345539
LPS IL-8	3.076281392	0.050392896	0.386345539
Neut Ab	2.311261007	0.104256724	0.685115613
SARS-S IFNg	1.791946281	0.171791175	0.871773325
CD4 naive%	1.777002177	0.17429047	0.871773325
CD8% CD3stim TNF	1.578524018	0.211228324	0.871773325
CD8% CD3stim TNF IFNg	1.448397015	0.239691283	0.871773325
CD8% CD3stim IL-2 IFNg	1.397323741	0.251904972	0.871773325
CD8% CD3stim IFNg	1.350151777	0.263749105	0.871773325
CD4% SARS-S IL-2 IFNg	1.275272544	0.283728087	0.871773325
CRP	1.273303081	0.28427391	0.871773325
LPS IL-6	1.161845781	0.316974506	0.886551874
CD4% CD3stim IFNg	1.062610135	0.349309788	0.886551874
LPS IFNg	0.88811448	0.414557475	0.886551874
CD4% CD3stim TNF IFNg	0.882930268	0.416675693	0.886551874
CD8% CD3stim TNF IL-2	0.852495031	0.429335705	0.886551874
CD8 EMRA%	0.733947239	0.482503785	0.886551874
CD8% SARS-S IL-2	0.66127088	0.518372364	0.886551874
CD4% CD3stim TNF IL-2	0.605297181	0.547842372	0.886551874
CD4 CM%	0.594760055	0.553578393	0.886551874
SARS-S TNF	0.562690028	0.571415733	0.886551874
CD8% CD3stim IL-2	0.539909719	0.584441129	0.886551874
CD8 CM%	0.500177493	0.607888259	0.886551874
CD4% SARS-S TNF IL-2	0.476768084	0.622149576	0.886551874
CD8 naive%	0.47488086	0.623314052	0.886551874
CD8% SARS-S IL-2 IFNg	0.472501536	0.624785335	0.886551874
SARS-S IL-6	0.44303565	0.643302077	0.886551874
CD4% CD3stim TNF	0.442221047	0.643821857	0.886551874
CD8% SARS-S TNF IFNg	0.411390106	0.663812413	0.886551874
CD4% SARS-S TNF	0.397191348	0.673230496	0.886551874
CD8 EM%	0.388853476	0.678824404	0.886551874
SARS-S IL-8	0.326272489	0.722351583	0.886551874
CD8% SARS-S TNF IL-2	0.314830072	0.730612785	0.886551874
CD4% CD3stim IL-2	0.312414662	0.73236894	0.886551874
LPS TNF	0.226823161	0.797458789	0.929748852

WB TNFa	0.213042162	0.808477262	0.929748852
CD4% SARS-S TNF IFNg	0.168778669	0.844928977	0.947969096
WB IL6	0.127331027	0.880580547	0.960642397
CD4% SARS-S IL-2	0.100642453	0.904345102	0.960642397
CD4% CD3stim IL-2 IFNg	0.08467435	0.918875337	0.960642397
WB IL8	0.035628401	0.965010707	0.986455389
CD8% SARS-S TNF	0.010166614	0.989885884	0.989885884

Supplementary Table 5: Immune correlates of pre-vaccine ratio on immune responses after SARS-CoV-2 vaccination, adjusting for other baseline and post-vaccination covariates.

F-statistic (mean sum of squares regression / mean sum of squares error) represents the degree of relatedness of each immunological parameter to pre-vaccine anti-S IgG/A/M antibody ratio. p values and q values (false discovery rate) derived using the quadratic regression for general linear models with adjustment for the following covariates: age, sex, BMI category, vaccine types, inter-vaccine days, days post second vaccine, vitamin D allocation.

Neut Ab = post-vaccine neutralising antibody; SARS-S = Stimulated with SARS-CoV-2 peptide; CD4% = percent of CD4+ T cells; CD8% = percent of CD8+ T cells; CM = central memory; EM = effector memory; EMRA = Effector memory re-expressing CD45RA; WB = whole blood stimulation assay; CRP = C Reactive protein; LPS = lipopolysaccharide stimulated; CD3stim= PBMCs stimulated with anti-CD3.

Analyte	t-statistic	p-value	q-value
**anti-S**	**-4.623263836**	**1.09E-05**	**0.000501258**
**CD4% SARS-S IFNg**	**-3.52959156**	**0.000621433**	**0.014292959**
CRP	-2.296160936	0.023672082	0.357012149
Neut Ab	-2.186182261	0.031044535	0.357012149
SARS-S IFNg	-2.068703175	0.041054111	0.359862698
WB LPS IL-8	2.010761976	0.046938613	0.359862698
CD8% CD3stim TNF IFNg	1.822531104	0.071247724	0.41029508
CD8% CD3stim IL-2 IFNg	1.802930832	0.074295023	0.41029508
CD8% CD3stim IFNg	1.766327143	0.080275124	0.41029508
CD8% CD3stim TNF	1.653887391	0.101165742	0.465362412
CD8% CD3stim TNF IL-2	1.476323724	0.142878523	0.597492005
CD4% SARS-S IL-2 IFNg	-1.275630713	0.204927995	0.785557313
CD4% CM	1.188724875	0.237255197	0.836120957
CD8% CD3stim IL-2	1.145887375	0.254471596	0.836120957
SARS-S TNF	1.043160558	0.299292715	0.917830992
CD8% SARS-S IL-2	0.973325014	0.332650223	0.921892899
CD4% EMRA	-0.925052464	0.357080119	0.921892899
CD8% SARS-S IL-2 IFNg	0.727716029	0.46842261	0.921892899
CD4 EM%	0.72763294	0.468473268	0.921892899
CD4% CD3stim IFNg	0.700625956	0.485101011	0.921892899
CD4% SARS-S TNF	0.679097116	0.498584877	0.921892899
SARS-S IL-6	0.651177108	0.516368422	0.921892899
CD8% SARS-S TNF IL-2	0.648652077	0.517993027	0.921892899
CD4% CD3stim TNF IFNg	0.643160105	0.5215358	0.921892899
CD8% SARS-S IFNg	-0.636750221	0.52568664	0.921892899
SARS-S IL-8	0.615340352	0.539674175	0.921892899
CD4% CD3stim TNF	0.596146405	0.552372747	0.921892899
CD4% CD3stim IL-2	0.551277578	0.58262587	0.921892899
WB LPS IL-6	0.496476501	0.620606018	0.921892899

WB IL8	0.494323015	0.622120613	0.921892899
CD4% naive	-0.493235677	0.62288598	0.921892899
CD8% SARS-S TNF	-0.464380443	0.643345865	0.921892899
CD8% EM	0.409217119	0.683221871	0.921892899
CD8% naive	-0.400262415	0.689784238	0.921892899
WB LPS TNF	0.372800857	0.710055366	0.921892899
CD4% SARS-S TNF IL-2	0.313317329	0.754667086	0.921892899
CD8 EMRA%	0.302048475	0.763218314	0.921892899
CD4% SARS-S IL-2	0.300995827	0.764018619	0.921892899
WB LPS IFNg	-0.26836741	0.788948121	0.921892899
CD8% SARS-S TNF IFNg	0.25186041	0.801645999	0.921892899
CD4% CD3stim TNF IL-2	0.162864655	0.870941042	0.977153365
CD4% SARS-S TNF IFNg	0.096983425	0.92292621	0.990078175
CD4% CD3stim IL-2 IFNg	0.076449193	0.93920861	0.990078175
CD8% CM	-0.06659586	0.947031298	0.990078175
WB TNFa	0.007108011	0.994342292	0.997269301
WB IL6	0.003430667	0.997269301	0.997269301

Supplementary Table 6: Immune correlates of pre-vaccine anti-S seronegativity (IgG/A/M antibody ratio <1) after SARS-CoV-2 vaccination, adjusting for other baseline and post-vaccination covariates.

t-statistic (regression co-efficient / standard deviation) represents the degree of relatedness of each immunological parameter to pre-vaccine anti-S seronegativity (IgG/A/M antibody ratio <1) compared to seropositivity (IgG/A/M antibody ratio ≥1). p values and q values (false discovery rate) derived using the t-test for general linear models with adjustment for the following covariates: age, sex, BMI category, vaccine types, inter-vaccine days, days post second vaccine, vitamin D allocation.

Neut Ab = post-vaccine neutralising antibody; SARS-S = Stimulated with SARS-CoV-2 peptide; CD4% = percent of CD4+ T cells; CD8% = percent of CD8+ T cells; CM = central memory; EM = effector memory; EMRA = Effector memory re-expressing CD45RA; WB = whole blood stimulation assay; CRP = C Reactive protein; LPS = lipopolysaccharide stimulated; CD3stim= PBMCs stimulated with anti-CD3.

Analyte	t-statistic	p-value	q-value
**anti-S**	**4.521405697**	**0.000015**	**0.000700526**
**Neut Ab**	**4.067364216**	**0.000088**	**0.002030986**
LPS TNF	2.164852619	0.032502356	0.498369455
CD4% CM	1.731644273	0.08606595	0.728300214
CD4% SARS-S IL-2	-1.62280798	0.107417134	0.728300214
CD8% EM	1.680470109	0.095628286	0.728300214
CD8% SARS-S IL-2	-1.607076764	0.110828293	0.728300214
CD4% naive	-1.469739676	0.144411041	0.743779567
CD4% CD3stim TNF IL-2	-1.408626676	0.16169121	0.743779567
CD8% naive	-1.460416079	0.146950573	0.743779567
CD4% CD3stim TNF	-1.348911285	0.180062418	0.752988293
CD4% CD3stim IL-2	-0.835657358	0.40511117	0.776463076
CD4% CD3stim IL-2 IFNy	-0.902766705	0.368570093	0.776463076
CD4% SARS-S IFNy	0.856334627	0.393625003	0.776463076
CD4% SARS-S TNF	-0.876020789	0.382877132	0.776463076
CD4% SARS-S TNF IFNy	-0.878779888	0.381385464	0.776463076
CD8% CM	1.059227824	0.291754954	0.776463076
CD8% CD3stim TNF	-1.173734069	0.242969483	0.776463076
CD8% SARS-S TNF IL-2	-1.008307815	0.315460952	0.776463076
WB LPS IFNy	-0.968313694	0.33495569	0.776463076
WB LPS IL-6	1.192560792	0.23553964	0.776463076
WB LPS IL-8	1.033303618	0.303668241	0.776463076
WB SARS-S IFNy	-0.891350746	0.374635055	0.776463076
WB SARS-S TNF	-1.114505649	0.267427148	0.776463076
CD4% SARS-S TNF IL-2	-0.769994915	0.44290964	0.793774022
WB IL6	-0.760306478	0.448654882	0.793774022
CD4% EM	0.64762646	0.518540194	0.795094965
CD8% CD3stim IL-2 IFNg	-0.671850979	0.50304986	0.795094965
CD8% SARS-S IL-2 IFNg	-0.726016045	0.469330016	0.795094965
WB IL8	-0.701652944	0.484338226	0.795094965
CD8% SARS-S TNF IFNy	-0.475702524	0.635204667	0.942561764
CD8% CD3stim TNF IL-2	-0.418027163	0.676720571	0.943307462
CRP	-0.420758009	0.674730932	0.943307462
CD4% CD3stim IFNy	-0.3179515	0.751108552	0.959749817
CD4% CD3stim TNF IFNy	-0.355161577	0.7231306	0.959749817
WB SARS-S IL-6	-0.331206918	0.741101613	0.959749817
CD8% CD3stim IL-2	-0.259221524	0.795935969	0.963501437
CD8% SARS-S TNF	0.267031133	0.789931912	0.963501437
CD4% SARS-S IL-2 IFNy	-0.190245017	0.849458485	0.967711782
CD8% EMRA	0.19088167	0.848960831	0.967711782
CD8% SARS-S IFNy	-0.173555002	0.862525719	0.967711782

CD4% EMRA	-0.032591127	0.974058143	0.985847417
CD8% CD3stim IFNy	-0.03478156	0.97231529	0.985847417
CD8% CD3stim TNF IFNy	-0.091835432	0.9269914	0.985847417
WB SARS-S IL-8	0.017777862	0.985847417	0.985847417
WB TNF	-0.046250455	0.963192267	0.985847417

Supplementary Table 7: Immune correlates of vaccine type after SARS-CoV-2 vaccination, adjusting for other baseline and post-vaccination covariates

t-statistic (regression co-efficient / standard deviation) represents magnitude of difference between vaccine types; a positive t-statistic indicates a higher value and negative a lower value of immunological parameter in participants receiving BNT162b2 as compared to ChAdOx1-nCoV-19. p values and q values (false discovery rate) derived using the t-test for general linear models with adjustment for the following covariates: age, sex, BMI category, inter-vaccine days, days post second vaccine, pre-vaccine SARS-CoV-2 sero-status, vitamin D allocation.

anti-S = anti-S IgG/A/M antibody ratio; Neut Ab = post-vaccine neutralising antibody; SARS-S = Stimulated with SARS-CoV-2 peptide; CD4% = percent of CD4+ T cells; CD8% = percent of CD8+ T cells; CM = central memory; EM = effector memory; EMRA = Effector memory re-expressing CD45RA; WB = whole blood stimulation assay; CRP = C Reactive protein; LPS = lipopolysaccharide stimulated; CD3stim= PBMCs stimulated with anti-CD3.

Analyte	F-statistic	p-value	q-value
CD4% SARS-S TNF IFNγ	5.194693089	0.006959193	0.160061445
CD8% SARS-S TNF	5.339371681	0.006097158	0.160061445
CD8% CD3stim TNF	4.467357635	0.013594212	0.208444579
CD4% CM	3.067777395	0.050453844	0.580219205
CD4% SARS-S IL-2 IFNγ	2.734654188	0.069253577	0.607720777
CD8% EM	2.529609919	0.084235324	0.607720777
CD8% CD3stim TNF IFNγ	2.432103634	0.092479249	0.607720777
CD4% EMRA	1.382207036	0.255272258	0.780251089
CD4% naive	1.402085185	0.250368713	0.780251089
CD4% CD3stim IL-2	1.199065924	0.305315644	0.780251089
CD4% CD3stim TNF IL-2	1.210202098	0.302005272	0.780251089
CD4% SARS-S IFNγ	1.353111506	0.262626413	0.780251089
CD4% SARS-S TNF IL-2	1.778921843	0.173556638	0.780251089
CD8% CM	1.241663337	0.292849029	0.780251089
CD8% naive	1.2514503	0.290058715	0.780251089
CD8% CD3stim IFNγ	1.570413232	0.212503503	0.780251089
CD8% SARS-S IL-2	1.214286208	0.300800393	0.780251089
WB TNF	1.23462379	0.294872922	0.780251089
CD4% EM	0.943771005	0.392230136	0.828076871
CD4% SARS-S IL-2	1.077064276	0.344095015	0.828076871
CD8% SARS-S IFNγ	0.954267919	0.388202363	0.828076871
WB LPS IL-6	0.933950782	0.396036765	0.828076871
CD4% CD3stim IL-2 IFNγ	0.536748707	0.586142284	0.837857191
CD4% CD3stim TNF	0.485101759	0.616921982	0.837857191
CD4% SARS-S TNF	0.593892992	0.553902891	0.837857191
CD8% CD3stim IL-2	0.706267893	0.495666831	0.837857191
CD8% CD3stim IL-2 IFNγ	0.828392446	0.439408789	0.837857191
CD8% CD3stim TNF IL-2	0.664264917	0.516667324	0.837857191
CD8% SARS-S IL-2 IFNγ	0.490405381	0.613686826	0.837857191
CD8% SARS-S TNF IL-2	0.527121723	0.591758709	0.837857191
Neut Ab	0.761065125	0.469569059	0.837857191
WB IL6	0.507131457	0.603596738	0.837857191
WB IL8	0.684206426	0.506586416	0.837857191
anti-S	0.481244534	0.61928575	0.837857191
WB SARS-S IFNγ	0.435780138	0.647851802	0.851462369
WB LPS IFNγ	0.386420488	0.680387106	0.869383525
WB SARS-S IL-8	0.330656797	0.719150642	0.894079177
CD4% CD3stim IFNγ	0.264310747	0.768212454	0.90609674
CRP	0.28804189	0.750283883	0.90609674
CD4% CD3stim TNF IFNγ	0.203312948	0.816323329	0.915874954

CD8% SARS-S TNF IFNγ	0.226675957	0.79754388	0.915874954
CD8% EMRA	0.173164219	0.841224224	0.921340817
WB SARS-S IL-6	0.122781418	0.88457585	0.924783844
WB SARS-S TNF	0.133890852	0.87482532	0.924783844
WB LPS IL-8	0.088489637	0.915376525	0.935718225
WB LPS TNF	0.050919335	0.950377315	0.950377315

Supplementary Table 8: Immune correlates of the number of days between vaccine one and two on immune responses after SARS-CoV-2 vaccination, adjusting for other baseline and post-vaccination covariates

F-statistic (mean sum of squares regression / mean sum of squares error) represents the degree of relatedness of each immunological parameter to the number of inter-vaccine days. p values and q values (false discovery rate) derived using the quadratic regression for general linear models with adjustment for the following covariates: age, sex, BMI category, vaccine types, days post second vaccine, pre-vaccine SARS-CoV-2 sero-status, vitamin D allocation.

anti-S = anti-S IgG/A/M antibody ratio; Neut Ab = post-vaccine neutralising antibody; SARS-S = Stimulated with SARS-CoV-2 peptide; CD4% = percent of CD4+ T cells; CD8% = percent of CD8+ T cells; CM = central memory; EM = effector memory; EMRA = Effector memory re-expressing CD45RA; WB = whole blood stimulation assay; CRP = C Reactive protein; LPS = lipopolysaccharide

Analyte	t-statistic	p-value	q-value
**WB SARS-S IL-6**	**5.816661358**	**6.81E-08**	**3.13E-06**
**WB SARS-S IL-8**	**5.316862106**	**6.16E-07**	**1.42E-05**
**WB SARS-S TNF**	**3.675478935**	**0.000379004**	**0.005811393**
Neut Ab	-2.54595637	0.012378562	0.142353462
CD8% CD3stim IFNγ	-2.34131575	0.021141218	0.163344184
CD8% CD3stim TNF IFNγ	-2.33826137	0.021305763	0.163344184
CD4% EMRA	2.202540159	0.029856457	0.196199577
WB TNF	2.07870698	0.040129141	0.230742562
CD8% CD3stim IL-2 IFNγ	-2.0018158	0.047933894	0.244995456
CD4% CM	-1.93632317	0.055568701	0.255616025
CD8% CD3stim TNF	-1.7974751	0.075190405	0.260196162
CD8% SARS-S TNF IL-2	-1.77872109	0.078235703	0.260196162
WB LPS IFNγ	1.772967577	0.079190136	0.260196162
anti-S	-1.85740244	0.066110782	0.260196162
WB LPS IL-8	-1.6853286	0.094952661	0.291188161
CD4% SARS-S IFNγ	-1.51195717	0.133606936	0.362787116
CD8% CD3stim IL-2	-1.46333373	0.146421128	0.362787116
CD8% SARS-S IL-2	-1.4230845	0.157733529	0.362787116
CD8% SARS-S TNF IFNγ	1.441500068	0.152476937	0.362787116
WB IL8	1.48661387	0.140171558	0.362787116
CD8% SARS-S TNF	1.257017016	0.211590477	0.443176692
WB LPS TNF	-1.25600946	0.21195407	0.443176692
CD8% CD3stim TNF IL-2	-1.09772456	0.274884366	0.512657872
CD8% SARS-S IL-2 IFNγ	-1.08917522	0.278618409	0.512657872
WB LPS IL-6	1.111456633	0.268959521	0.512657872
CD4% SARS-S IL-2	-0.82717258	0.410051507	0.723345024
WB IL6	0.801700234	0.424572079	0.723345024
CD4% EM	0.737711966	0.46236686	0.759602699
CD4% SARS-S TNF	0.602979541	0.547847707	0.868999812
CD4% CD3stim IL-2	-0.43791673	0.662363631	0.897725744
CD4% CD3stim TNF	0.409460247	0.683052197	0.897725744
CD4% CD3stim TNF IL-2	-0.42724279	0.670094318	0.897725744
CD8% CM	-0.41620389	0.678126783	0.897725744
CD8% EM	-0.50168902	0.616956977	0.897725744
CD8% SARS-S IFNγ	0.467321068	0.641257333	0.897725744
CD4% CD3stim IL-2 IFNγ	-0.35103887	0.726276186	0.899126403
CD4% SARS-S TNF IL-2	-0.30959654	0.757493253	0.899126403
CD8% naive	0.303260773	0.76230282	0.899126403
WB SARS-S IFNγ	0.338321298	0.735809726	0.899126403
CRP	-0.25144184	0.801973502	0.922269527
CD4% naive	0.119311832	0.905260709	0.925377614

CD4% CD3stim IFNγ	-0.15342098	0.878366475	0.925377614
CD4% CD3stim TNF IFNγ	-0.12735347	0.89890894	0.925377614
CD4% SARS-S TNF IFNγ	0.166349515	0.868208013	0.925377614
CD8% EMRA	-0.13402428	0.893644905	0.925377614
CD4% SARS-S IL-2 IFNγ	-0.09183755	0.92700549	0.92700549

Supplementary Table 9: Immune correlates with the number of days between the second vaccine and day of blood draw to measure on immune responses after SARS-CoV-2 vaccination, adjusting for other baseline and post-vaccination covariates

t-statistic (regression co-efficient / standard deviation) represents magnitude of relationship between each immunological parameter to the number of days after the second vaccine and blood sampling. p values and q values (false discovery rate) derived using linear regression for general linear models with adjustment for the following covariates: age, sex, BMI category, vaccine types, pre-vaccine SARS-CoV-2 sero-status, inter-vaccine days, vitamin D allocation.

anti-S = anti-S IgG/A/M antibody ratio; Neut Ab = post-vaccine neutralising antibody; SARS-S = Stimulated with SARS-CoV-2 peptide; CD4% = percent of CD4+ T cells; CD8% = percent of CD8+ T cells; CM = central memory; EM = effector memory; EMRA = Effector memory re-expressing CD45RA; WB = whole blood stimulation assay; CRP = C Reactive protein; LPS = lipopolysaccharide stimulated; CD3stim= PBMCs stimulated with anti-CD3.

Analyte	t-statistic	p-value	q-value
**CD8% naive**	**-6.228470802**	**1.04E-08**	**4.78E-07**
**CD8% EM**	**2.97858429**	**0.003611396**	**0.042298032**
**CD8% EMRA**	**2.972481966**	**0.00367809**	**0.042298032**
**anti-S**	**-3.139087915**	**0.002211252**	**0.042298032**
**CD4% SARS-S IL-2 IFNγ**	**-2.549304485**	**0.012267475**	**0.09590231**
**CD4% SARS-S TNF IL-2**	**-2.542059422**	**0.012508997**	**0.09590231**
CD4% CD3stim IL-2	-2.006546497	0.047418833	0.311609471
CD4% CD3stim TNF IL-2	-1.905206323	0.05954217	0.342367477
CD8% CM	1.791730642	0.076112588	0.389019895
CD4% EM	1.181297064	0.240205144	0.587892344
CD4% naive	-1.288634181	0.20041226	0.587892344
CD4% SARS-S TNF IFNγ	-1.348785639	0.180363843	0.587892344
CD8% CD3stim IL-2 IFNγ	-1.17469573	0.242825099	0.587892344
CD8% CD3stim TNF	1.303633928	0.195264679	0.587892344
CD8% CD3stim TNF IL-2	-1.454822659	0.148759184	0.587892344
CD8% SARS-S TNF IFNγ	1.19952631	0.23307542	0.587892344
Neut Ab	-1.24086082	0.217476018	0.587892344
WB SARS-S IL-8	-1.446853518	0.15097449	0.587892344
WB SARS-S TNF	-1.356435537	0.177926461	0.587892344
CD4% SARS-S IFNγ	1.004557252	0.317464248	0.7014113
CD8% CD3stim IL-2	-0.976980507	0.330867861	0.7014113
CD8% CD3stim TNF IFNγ	0.937478244	0.35070565	0.7014113
WB LPS TNF	0.962249219	0.338178038	0.7014113
CD4% CM	0.872027755	0.385221441	0.738341096
WB LPS IL-8	-0.833280206	0.406614865	0.748171351
CD8% SARS-S TNF	0.782918513	0.435471371	0.770449349
CD8% SARS-S IFNγ	0.747615278	0.456395709	0.77756306
CD8% CD3stim IFNγ	0.691912115	0.490549318	0.778112711
WB SARS-S IL-6	-0.710655749	0.478903673	0.778112711
CD8% SARS-S IL-2 IFNγ	-0.649149895	0.517686388	0.793785794
WB IL8	-0.590743661	0.555986234	0.825011832
CRP	0.549275875	0.584005033	0.839507235
CD4% CD3stim TNF	-0.437128425	0.662933331	0.924088886
WB LPS IL-6	0.359537393	0.719929186	0.9268921
SARS-S IFNγ	-0.352218807	0.725393817	0.9268921
WB IL6	-0.405275166	0.686115808	0.9268921
CD4% EMRA	0.277177006	0.782199713	0.938712658
CD8% SARS-S IL-2	0.259373099	0.79586508	0.938712658
WB TNF	0.304923832	0.761039459	0.938712658
CD4% CD3stim IL-2 IFNγ	-0.153126284	0.878598276	0.962274302
CD8% SARS-S TNF IL-2	-0.156635746	0.875838541	0.962274302

WB LPS IFNγ	0.200240687	0.841687181	0.962274302
CD4% CD3stim IFNγ	-0.050171085	0.960083218	0.980254766
CD4% CD3stim TNF IFNγ	-0.047316428	0.962352658	0.980254766
CD4% SARS-S IL-2	0.024809688	0.980254766	0.980254766
CD4% SARS-S TNF	-0.032009501	0.97452642	0.980254766

Supplementary Table 10: Immune correlates between age and immune responses after SARS-CoV-2 vaccination, adjusting for other baseline and post-vaccination covariates

t-statistic (regression co-efficient / standard deviation) represents magnitude of relationship between each immunological parameter to year of age. p values and q values (false discovery rate) derived using linear regression for general linear models with adjustment for the following covariates: sex, BMI category, vaccine types, inter-vaccine days, days post second vaccine, pre-vaccine SARS-CoV-2 sero-status, vitamin D allocation.

anti-S = anti-S IgG/A/M antibody ratio; Neut Ab = post-vaccine neutralising antibody; SARS-S = Stimulated with SARS-CoV-2 peptide; CD4% = percent of CD4+ T cells; CD8% = percent of CD8+ T cells; CM = central memory; EM = effector memory; EMRA = Effector memory re-expressing CD45RA; WB = whole blood stimulation assay; CRP = C Reactive protein; LPS = lipopolysaccharide

Analyte	F-statistic	p-value	q-value
**CD4% SARS-S TNF**	**13.34089279**	**2.01E-07**	**9.24E-06**
**CD8% SARS-S TNF**	**9.893043518**	**8.65E-06**	**0.000198858**
CD4% SARS-S IL-2	3.781968117	0.012769521	0.155897819
CRP	3.734163523	0.013556332	0.155897819
CD4% EM	2.265696049	0.085278286	0.435866793
CD8% CM	2.331936359	0.07851803	0.435866793
CD8% EMRA	2.416109085	0.070686109	0.435866793
CD8% CD3stim IL-2	2.436052084	0.068946709	0.435866793
CD8% SARS-S IL-2	2.339568377	0.077773932	0.435866793
CD4% CM	1.923565388	0.130395817	0.487805718
CD4% SARS-S TNF IL-2	1.736675382	0.164126928	0.487805718
CD8% CD3stim IFNγ	1.745974183	0.162265564	0.487805718
CD8% CD3stim TNF IL-2	2.028518438	0.114515798	0.487805718
CD8% SARS-S IL-2 IFNγ	1.898503065	0.134494113	0.487805718
WB LPS IFNγ	1.84949255	0.142873201	0.487805718
WB LPS IL-6	1.660010099	0.180276026	0.487805718
WB LPS TNF	1.694372058	0.172857538	0.487805718
CD4% EMRA	1.499236465	0.219217646	0.504200585
CD8% CD3stim IL-2 IFNγ	1.570018411	0.20117689	0.504200585
CD8% CD3stim TNF IFNγ	1.501374364	0.218650964	0.504200585
CD4% naive	1.317798018	0.272678919	0.507418637
CD4% CD3stim IFNγ	1.308351755	0.275770999	0.507418637
CD4% CD3stim IL-2 IFNγ	1.437575936	0.236171489	0.507418637
CD4% CD3stim TNF IFNγ	1.368216634	0.256712754	0.507418637
CD8% SARS-S TNF IL-2	1.347492218	0.263167276	0.507418637
CD8% CD3stim TNF	1.065312862	0.367237763	0.64972835
CD4% SARS-S IL-2 IFNγ	1.029613495	0.382752401	0.652096684
anti-S	0.956532121	0.416307417	0.683933614
WB SARS-S TNF	0.837654352	0.476235709	0.755408367
CD8% naive	0.733924806	0.534101323	0.792537448
WB TNF	0.750576198	0.52445981	0.792537448
CD4% CD3stim TNF	0.68251729	0.564710995	0.799612798
CD4% SARS-S IFNγ	0.623096943	0.601649702	0.799612798
CD8% EM	0.61251086	0.608401042	0.799612798
WB SARS-S IL-8	0.62098521	0.602992433	0.799612798
CD8% SARS-S TNF IFNγ	0.504633486	0.679932034	0.845320907
WB LPS IL-8	0.523700774	0.666947847	0.845320907
CD4% SARS-S TNF IFNγ	0.380030721	0.767595065	0.882734324
CD8% SARS-S IFNγ	0.388651192	0.76140946	0.882734324
WB IL6	0.424523562	0.735822283	0.882734324
CD4% CD3stim IL-2	0.292669058	0.830610472	0.931904432

CD4% CD3stim TNF IL-2	0.183677852	0.907277768	0.93718602
Neut Ab	0.144979641	0.932683726	0.93718602
WB SARS-S IFNγ	0.210271448	0.889085492	0.93718602
WB SARS-S IL-6	0.242032945	0.86682437	0.93718602
WB IL8	0.137849733	0.93718602	0.93718602

Supplementary Table 11: Immune correlates between BMI category and immune responses after SARS-CoV-2 vaccination, adjusting for other baseline and post-vaccination covariates

F-statistic (mean sum of squares regression / mean sum of squares error) represents the degree of relatedness of each immunological parameter to BMI category. p values and q values (false discovery rate) derived using ANOVA for general linear models with adjustment for the following covariates: age, sex, vaccine types, inter-vaccine days, days post second vaccine, pre-vaccine SARS-CoV-2 sero-status, vitamin D allocation.

anti-S = anti-S IgG/A/M antibody ratio; Neut Ab = post-vaccine neutralising antibody; SARS-S = Stimulated with SARS-CoV-2 peptide; CD4% = percent of CD4+ T cells; CD8% = percent of CD8+ T cells; CM = central memory; EM = effector memory; EMRA = Effector memory re-expressing CD45RA; WB = whole blood stimulation assay; CRP = C Reactive protein; LPS = lipopolysaccharide stimulated; CD3stim= PBMCs stimulated with anti-CD3.

Analyte	t-statistic	p-value	q-value
**WB LPS IL-6**	**4.158295155**	**6.27E-05**	**0.002489318**
**CD8% EM**	**4.012705803**	**0.000108231**	**0.002489318**
**CD8% naive**	**-3.182844162**	**0.001883947**	**0.02888719**
**CD4% CM**	**3.01518178**	**0.003172075**	**0.036478867**
**WB LPS IL-8**	**2.913638115**	**0.004307056**	**0.039624915**
**CD4% EM**	**2.567760468**	**0.011541032**	**0.079635906**
**CD4% naive**	**-2.549779177**	**0.012118507**	**0.079635906**
CD8% CM	2.294992447	0.023579684	0.135583181
WB LPS TNF	1.751663685	0.082544731	0.42189529
CD4% EMRA	-1.522587419	0.130655571	0.441795653
CD4% SARS-S IFNγ	-1.592615604	0.114039998	0.441795653
CD4% SARS-S TNF IFNγ	1.447651625	0.150483264	0.441795653
WB SARS-S IL-6	1.616152287	0.108849931	0.441795653
WB SARS-S IL-8	1.489691854	0.139091161	0.441795653
WB SARS-S TNF	1.490472198	0.138886246	0.441795653
anti-S	1.436341166	0.153668053	0.441795653
CD8% SARS-S IL-2	-1.355089664	0.178091714	0.481895225
CD4% SARS-S IL-2 IFNγ	1.222740889	0.223971666	0.542810819
CD8% CD3stim TNF IFNγ	1.22212255	0.224204469	0.542810819
CD8% CD3stim IFNγ	1.164691687	0.246596752	0.567172529
CD4% SARS-S IL-2	-1.080163479	0.282369432	0.618523518
CD8% CD3stim TNF	1.042273998	0.299509431	0.626246992
CD4% CD3stim IFNγ	0.588249743	0.557538563	0.802987041
CD4% CD3stim IL-2	-0.55022037	0.583254232	0.802987041
CD4% CD3stim TNF IFNγ	0.755102396	0.451758516	0.802987041
CD4% SARS-S TNF	-0.593918622	0.553753652	0.802987041
CD4% SARS-S TNF IL-2	0.820012748	0.413935175	0.802987041
CD8% SARS-S TNF	-0.560191274	0.576457882	0.802987041
CD8% SARS-S TNF IFNγ	0.664710104	0.507590186	0.802987041
Neut Ab	0.739851773	0.460924267	0.802987041
WB SARS-S IFNγ	-0.604854286	0.546488507	0.802987041
WB IL6	0.535274148	0.593512161	0.802987041
WB IL8	0.612185717	0.541644781	0.802987041
WB TNF	0.751627922	0.45383747	0.802987041
WB LPS IFNγ	0.463643044	0.643795355	0.846131038
CD4% CD3stim TNF IL-2	-0.409734994	0.682776054	0.847087595
CD8% EMRA	0.397966474	0.691405752	0.847087595
CD8% CD3stim IL-2	-0.357531309	0.721360997	0.847087595
CD8% CD3stim TNF IL-2	-0.317172229	0.751698191	0.847087595
CD8% SARS-S IFNγ	0.367500424	0.713933175	0.847087595
CRP	0.312795132	0.755012857	0.847087595

CD8% SARS-S IL-2 IFNγ	-0.281562924	0.77879364	0.852964463
CD8% CD3stim IL-2 IFNγ	-0.245385736	0.806602876	0.862877496
CD4% CD3stim IL-2 IFNγ	0.164409906	0.869702196	0.889028912
CD8% SARS-S TNF IL-2	0.181521699	0.856283308	0.889028912
CD4% CD3stim TNF	0.032635368	0.97402294	0.97402294

Supplementary Table 12: Immune correlates between Sex and immune responses after SARS-CoV-2 vaccination, adjusting for other baseline and post-vaccination covariates

t-statistic (regression co-efficient / standard deviation) represents magnitude of difference between sexes; a positive t-statistic indicates a higher value and negative a lower value of immunological parameter in males participants compared to female. p values and q values (false discovery rate) derived using the t-test for general linear models with adjustment for the following covariates: age, BMI category, vaccine types, inter-vaccine days, days post second vaccine, pre-vaccine SARS-CoV-2 sero-status, vitamin D allocation.

anti-S = anti-S IgG/A/M antibody ratio; Neut Ab = post-vaccine neutralising antibody; SARS-S = Stimulated with SARS-CoV-2 peptide; CD4% = percent of CD4+ T cells; CD8% = percent of CD8+ T cells; CM = central memory; EM = effector memory; EMRA = Effector memory re-expressing CD45RA; WB = whole blood stimulation assay; CRP = C Reactive protein; LPS = lipopolysaccharide stimulated; CD3stim= PBMCs stimulated with anti-CD3.
