# Supplementary material for: Influence of individuals’ determinants including vaccine type on cellular and humoral responses to SARS-CoV-2 vaccination
Source: NPJ Vaccines. 2024 May 22;9:87. doi: 10.1038/s41541-024-00878-0 (PMC11111746; doi:10.1038/s41541-024-00878-0)
Supplement: Supplementary file 2 — REPORTING SUMMARY [file 41541_2024_878_MOESM2_ESM.pdf]

nature portfolio

Corresponding author(s): Emma S Chambers

Last updated by author(s): Feb 15, 2024

Reporting Summary

Nature Portfolio wishes to improve the reproducibility of the work that we publish. This form provides structure for consistency and transparency in reporting. For further information on Nature Portfolio policies, see our Editorial Policies and the Editorial Policy Checklist.

Statistics

For all statistical analyses, confirm that the following items are present in the figure legend, table legend, main text, or Methods section.

n/a	Confirmed	
☐	☒	The exact sample size (*n*) for each experimental group/condition, given as a discrete number and unit of measurement
☒	☐	A statement on whether measurements were taken from distinct samples or whether the same sample was measured repeatedly
☐	☒	The statistical test(s) used AND whether they are one- or two-sided<br>*Only common tests should be described solely by name; describe more complex techniques in the Methods section.*
☐	☒	A description of all covariates tested
☐	☒	A description of any assumptions or corrections, such as tests of normality and adjustment for multiple comparisons
☐	☒	A full description of the statistical parameters including central tendency (e.g. means) or other basic estimates (e.g. regression coefficient) AND variation (e.g. standard deviation) or associated estimates of uncertainty (e.g. confidence intervals)
☐	☒	For null hypothesis testing, the test statistic (e.g. *F*, *t*, *r*) with confidence intervals, effect sizes, degrees of freedom and *P* value noted<br>*Give P values as exact values whenever suitable.*
☒	☐	For Bayesian analysis, information on the choice of priors and Markov chain Monte Carlo settings
☒	☐	For hierarchical and complex designs, identification of the appropriate level for tests and full reporting of outcomes
☐	☒	Estimates of effect sizes (e.g. Cohen's *d*, Pearson's *r*), indicating how they were calculated

Our web collection on statistics for biologists contains articles on many of the points above.

Software and code

Policy information about availability of computer code

Data collection	n/a
Data analysis	Principle component analysis (PCA) and general linear modelling (GLM) was conducted using Qlucore Omics Explorer 3.7 (Qlucore AB, Lund, Sweden).

For manuscripts utilizing custom algorithms or software that are central to the research but not yet described in published literature, software must be made available to editors and reviewers. We strongly encourage code deposition in a community repository (e.g. GitHub). See the Nature Portfolio guidelines for submitting code & software for further information.

Data

Policy information about availability of data

All manuscripts must include a data availability statement. This statement should provide the following information, where applicable:

- Accession codes, unique identifiers, or web links for publicly available datasets
- A description of any restrictions on data availability
- For clinical datasets or third party data, please ensure that the statement adheres to our policy

The data that support the findings of this study are available on request from the corresponding author, [ES Chambers]. The data are not publicly available due to it containing information that could compromise the privacy of research participants.

Research involving human participants, their data, or biological material

Policy information about studies with human participants or human data. See also policy information about sex, gender (identity/presentation), and sexual orientation and race, ethnicity and racism.

Reporting on sex and gender	This was a population based study that was carried out on both men and women.Detailed breakdown of participant characteristics are found in table 1.
Reporting on race, ethnicity, or other socially relevant groupings	This was a population based study that was carried out all individuals that volunteered for the study. Detailed breakdown of participant characteristics are found in table 1.
Population characteristics	To identify demographic and technical factors associated with humoral and cellular responses to COVID-19 vaccination we first performed univariate analysis of ten factors: age, sex, ethnicity, general health category, vaccine type, number of days between 1st and 2nd vaccination [inter-vaccine days], days post second vaccine, pre-vaccine SARS-CoV-2 sero-status, BMI value, and BMI category. This analysis identified seven factors which had a significant association with cellular and/or humoral immune responses: age, sex, pre-vaccine SARS-CoV-2 sero-status, BMI category, vaccine type, inter-vaccine days, and days post second vaccine (Supplementary Tables 2a-i). These seven factors were adjusted for in subsequent analyses to identify independent associations
Recruitment	This was a population based study - were individuals volunteered for the research study.
Ethics oversight	The trial was sponsored by Queen Mary University of London, approved by the Queens Square Research Ethics Committee, London, UK (ref 20/HRA/5095) and registered with ClinicalTrials.gov (NCT04579640) on 8 October 2020.

Note that full information on the approval of the study protocol must also be provided in the manuscript.

Field-specific reporting

Please select the one below that is the best fit for your research. If you are not sure, read the appropriate sections before making your selection.

☒ Life sciences ☐ Behavioural & social sciences ☐ Ecological, evolutionary & environmental sciences

For a reference copy of the document with all sections, see nature.com/documents/nr-reporting-summary-flat.pdf

Life sciences study design

All studies must disclose on these points even when the disclosure is negative.

Sample size	This was a population based study .
Data exclusions	Of the 127 participants with acquired data, 12 were excluded from statistical analysis (two due to haematological malignancy with an absence of CD4+ T cells, four due to immunodeficiency, three for being on immunosuppressive medication, one as having reported worst health and two who were identified as outliers on PCA (Supplementary Figure 3).
Replication	n/a
Randomization	All participants gave blood at only one time point.
Blinding	The participant information was blinded to the lab scientists who were carrying out the experiments.

Reporting for specific materials, systems and methods

We require information from authors about some types of materials, experimental systems and methods used in many studies. Here, indicate whether each material, system or method listed is relevant to your study. If you are not sure if a list item applies to your research, read the appropriate section before selecting a response.

Materials & experimental systems

n/a	Involved in the study
☒	☐ Antibodies
☒	☐ Eukaryotic cell lines
☒	☐ Palaeontology and archaeology
☒	☐ Animals and other organisms
☒	☐ Clinical data
☒	☐ Dual use research of concern
☒	☐ Plants

Methods

n/a	Involved in the study
☒	☐ ChIP-seq
☐	☒ Flow cytometry
☒	☐ MRI-based neuroimaging

Plants

Seed stocks

Report on the source of all seed stocks or other plant material used. If applicable, state the seed stock centre and catalogue number. If plant specimens were collected from the field, describe the collection location, date and sampling procedures.

Novel plant genotypes

Describe the methods by which all novel plant genotypes were produced. This includes those generated by transgenic approaches, gene editing, chemical/radiation-based mutagenesis and hybridization. For transgenic lines, describe the transformation method, the number of independent lines analyzed and the generation upon which experiments were performed. For gene-edited lines, describe the editor used, the endogenous sequence targeted for editing, the targeting guide RNA sequence (if applicable) and how the editor was applied.

Authentication

Describe any authentication procedures for each seed stock used or novel genotype generated. Describe any experiments used to assess the effect of a mutation and, where applicable, how potential secondary effects (e.g. second site T-DNA insertions, mosiacism, off-target gene editing) were examined.

Flow Cytometry

Plots

Confirm that:

☒ The axis labels state the marker and fluorochrome used (e.g. CD4-FITC).

☒ The axis scales are clearly visible. Include numbers along axes only for bottom left plot of group (a 'group' is an analysis of identical markers).

☒ All plots are contour plots with outliers or pseudocolor plots.

☒ A numerical value for number of cells or percentage (with statistics) is provided.

Methodology

Sample preparation

Cells were cell surface stained for CD3 (HIT3a), CD4 (RPA-T4), CD8 (SK1), CD27 (O232), CD45RA (HI100) and Zombie NIRTM viability dye (Biolegend, San Diego, CA, USA) in the presence of Brilliant Buffer (BD Biosciences). Cells were washed and then fixed in Intracellular Fixation Buffer (eBioscience), permeabilised in eBioscience Permeablization Buffer and stained for intracellular IL-2 (JES6-5H4), IFN-γ (4S.B3) and TNF (Mab11, Biolegend).

Instrument

ACEA Novocyte 3000

Software

Samples were collected on NOVOEXPRESS and analysed using FlowJo version 10

Cell population abundance

samples were not sorted

Gating strategy

gating strategy is provided in supplementary figures 1 and 2

☒ Tick this box to confirm that a figure exemplifying the gating strategy is provided in the Supplementary Information.
